# Immune checkpoint inhibitor-based combinatory and alternative strategies for immune treatment of lung cancers

**DOI:** 10.3389/fimmu.2026.1878931

**Published:** 2026-08-10

**Authors:** Jun-Young Park, Choong-Hwan Kwak, Yu-Chan Chang, Cheorl-Ho Kim

**Affiliations:** 1 Molecular and Cellular Glycobiology Unit, Department of Biological Sciences, SungKyunKwan University, Suwon, Gyunggi-Do, Republic of Korea; 2 Environmental Diseases Research Center, Korea Research Institute of Bioscience and Biotechnology, Daejeon, Republic of Korea; 3 Department of Brewing, Dong-Eui Institute of Technology, Busan, Republic of Korea; 4 Department of Biomedicine Imaging and Radiological Sciences, National Yang Ming Chiao Tung University, Taipei, Taiwan

**Keywords:** anti-PD-l/PD-LI therapy, cancer cells, ICI, KRAS, therapy-resistant tumors

## Abstract

Immune checkpoint inhibitors (ICIs), particularly antibodies targeting PD-1/PD-L1 and CTLA-4, have reshaped the treatment landscape of lung cancers, most notably non-small cell lung cancer (NSCLC). Nevertheless, only a subset of patients achieve durable benefit due to primary and acquired resistance that arises from tumor-intrinsic factors (e.g., oncogenic drivers and adaptive signaling), tumor-extrinsic determinants in the tumor microenvironment (TME) (e.g., impaired T-cell infiltration and immunosuppressive myeloid populations), and the dynamic evolution of biomarkers. Accordingly, current clinical and translational efforts in lung cancer focus on rational combination strategies—ICIs with chemotherapy, radiotherapy, and targeted agents (including RTK- and KRAS-pathway inhibitors)—as well as alternative approaches that modulate antigen presentation, myeloid regulation, and cancer stemness. In this review, we define a lung cancer–centered scope and summarize (i) established and emerging ICI-based regimens in lung cancer, (ii) mechanisms of resistance relevant to NSCLC and SCLC, (iii) biomarker integration for patient selection and monitoring, and (iv) future directions to optimize efficacy and safety through combinatory and alternative immunotherapeutic strategies.

## 1 Introduction

The immune system maintains homeostasis by surveying pathogenic invaders and transformed cells through continuous communication between host cells and their environments. Immune checkpoints are physiological regulators that limit excessive T-cell activation and maintain self-tolerance. In cancer, tumors frequently exploit these pathways—most prominently the PD-1/PD-L1 axis and CTLA-4—to suppress anti-tumor immunity within the tumor microenvironment (TME) (1). In cancer, immune evasion is promoted by genetic instability and tumor evolution, which generate heterogeneous subclones and neoantigen landscapes (2). Importantly for lung cancer, smoking-associated mutational processes and intratumoral heterogeneity can shape tumor mutation burden, antigenicity, and responsiveness to IC blockade. Mechanistically, tumors may reduce antigen presentation (e.g., via MHC-I alterations), remodel surface antigens, and establish an immunosuppressive microenvironment to escape immune surveillance (3). Clinically, monoclonal antibodies that block PD-1 (e.g., Nivolumab/Opdivo, Pembrolizumab/Keytruda), PD-L1 (e.g., Durvalumab/Imfinzi, Atezolizumab/Tecentriq), and CTLA-4 (e.g., Ipilimumab) have become core immunotherapeutic agents, either as monotherapy or in combination regimens; representative approvals are summarized in **Table 1**. Building on this shared framework, the key questions addressed throughout this review are how to extend response durability, overcome resistance mechanisms, and rationally combine ICIs with other modalities.

**Table 1:** FDA-approved ICI.

Drug name	Cancer type	Target	Approval [ref]
Pembrolizumab	Malignant melanoma/BRAF V600 mutation	PD-1	2014 (46)
Nivolumab/Pembrolizumab	Malignant HNC and SCC	PD-1	2016 (47)
Nivolumab	Melanoma	PD-1	2014 (51)
Pembrolizumab	HNC	PD-1	2016 (47)
	Melanoma	PD-1	2015 (57)
Pembrolizumab/Nivolumab	HNC and SCC	PD-1	2016 (47)
Cemiplimab	SCC	PD-1	2018 (48)
Ipilimumab	Metastatic melanoma	CTLA-4	2011 (48)
Ipilimumab	Melanoma	CTLA-4	2015 (56)
Ipilimumab+Nivolumab	Metastatic melanoma	CTLA-4+ PD1	2015 (50)
Avelumab	Metastatic Merkel cell carcinoma	PD-L1	2015 (53)

HNC, head and neck cancer; SCC, squamous cell carcinoma.

Beyond these canonical checkpoints, additional inhibitory and stimulatory receptors (e.g., LAG-3, TIM-3, TIGIT, and others) also shape anti-tumor immunity and are being actively investigated as therapeutic targets. Accordingly, ICI-based strategies increasingly consider not only checkpoint selection but also T-cell priming, antigen presentation, and the balance of suppressive versus effector immune populations within the TME. Mechanistically, checkpoint blockade can reinvigorate exhausted T cells and restore cytotoxic activity; however, its clinical impact varies substantially because tumors differ in antigenicity, interferon signaling, stromal architecture, and immune composition. Accordingly, ICI responsiveness is increasingly viewed as an emergent property of both tumor-intrinsic determinants (e.g., oncogenic signaling, antigen presentation defects) and tumor-extrinsic constraints (e.g., suppressive myeloid populations and dysfunctional antigen-presenting cells) (3). This perspective motivates biomarker-driven treatment selection and the design of combinations that convert “cold” tumors into “hot” immune ecosystems.

In lung cancer, these principles are particularly relevant because mutational processes, intratumoral heterogeneity, and therapy-induced evolution can dynamically reshape the immune landscape. Even when PD-L1 expression or tumor mutation burden suggests benefit, resistance may arise through adaptive pathway rewiring, impaired T-cell infiltration, or expansion of immunosuppressive myeloid-derived suppressor cells and tumor-associated macrophages. Therefore, effective lung cancer immunotherapy increasingly requires coordinated strategies that address both the cancer cell and the TME, rather than isolated inhibition of a single checkpoint.

Beyond checkpoint blockade alone, precision-oncology approaches now integrate targeted therapy, radiotherapy, and chemotherapy with ICIs to amplify tumor antigen release, enhance immune priming, and reduce immune escape. In parallel, emerging work links epigenetic and stem-like tumor programs to immune resistance, providing additional therapeutic entry points. These considerations collectively support a modular view of ICI-based treatment in which the optimal regimen is selected based on actionable drivers, immune contexture, and longitudinal biomarkers.

To operationalize this strategy, molecular profiling and liquid-biopsy technologies have expanded the ability to track tumor evolution and to match patients to rational combinations (4, 5). In lung cancer, next-generation sequencing of tumor tissue and circulating tumor DNA can identify oncogenic drivers, resistance alterations, and immunotherapy-relevant features (e.g., pathway activation patterns and genomic correlates of immune exclusion). These data increasingly inform trial design and clinical decision making, particularly when integrating ICIs with receptor tyrosine kinase (RTK) inhibitors or downstream pathway modulators.

With this background, the remainder of this review is organized to maintain a lung cancer–centered narrative: we first outline the clinical logic of ICI-based regimens, then discuss mechanistic categories of resistance, and finally summarize combination and alternative strategies that target oncogenic signaling, the TME, and therapy-resistant tumor states, with an emphasis on biomarker-guided translation.

As another approach to target the tumor-specific alterations apart from ICI, TKIs specific to the active gene mutations and rearrangements have been designed and developed. Also, to improve the inherent efficacy and resistance issues of TKIs, the liquid biopsy NGS of exosomal blood ctDNA as a ctcDNA has been diagnostically approved by the Food and Drug Administration (FDA) to detect quantitative and qualitative extrachromosomal DNA (ecDNA) from NSCLC (5, 6). It has been known that the ecDNAs of the NSCLC include ALK, BRAF, DDR1/2, EGFR, ERBB2, Kirsten rat sarcoma (KRAS), MNNG HOS transforming gene, NTRK, mesenchymal epithelial transition (MET), RET, ROS1, and SOS Ras/Rac guanine nucleotide exchange factor 1 (SOS1) as well as PD-L1 and tumor mutation burden expression, covering gene amplifications, gene rearrangements, point mutations, gene deletions, epigenetic modifications, gene methylation alterations, gene insertions, gene fusions, and copy number alterations in blood and tissues (7, 8). To date, EGFR and BRAF mutations, ALK gene rearrangements, and ROS1 fusion genes have been identified (9).

Tumor-targeting inhibitors have been combined to overcome their inherent limitations by several strategies adopted from conventional signaling inhibitors. For example, therapy-resistant EGFR mutations have been alternatively targeted by allosteric (ATP non-competitive) inhibitors, which differ from conventional EGFR TKIs. The EGFR allosteric TKIs recognize a specific, distinct EGFR site that is different from the existing ATP-competitive inhibitors. These EGFR allosteric TKIs call for next-generation allosteric inhibitors. Such next-generation allosteric inhibitors against the conventional therapy-resistant EGFR mutants have been combined for effective therapy in NSCLC. Cancer stem cells (CSC) have also been associated with tumor resistance aspects.

Finally, there is a recently recognized issue why the above tumor treatments are not as efficient rather than expected. A difficult reason is explained by the CSC in terms of tumor resistance aspects. CSC tumor phenotypes are regulated by Sonic Hedgehog (Shh) or Notch-related signaling pathways. CSC can be targeted by the transmembrane (TM) protein smoothened (SMO) known as the hedgehog (HH) pathway receptor (HHR). The HHR SMO inhibitors are used as therapeutic agents. Therefore, to overcome the CSC issues, future studies on these signal blocking and cancer cell death are required. Representative CSC-modulating drugs such as Vismodegib, Sonidegib, and Glasdegib, however, exhibit severe adverse effects but effective for only based cell carcinomas. For various tumor therapeutic targets, serine hydroxymethyltransferase 1 (SHMT1) siRNA can silence to block DNA synthesis in tumor cells. Anti-cancer antibiotics, carbonic anhydrase 9 (CAIX) targeting peptides, importin β- and claudin 3 (CLDN3)–targeting inhibitors enhance the therapeutic potential against tumors.

Here, we review ICI-based combinatory and alternative strategies for lung cancers, with a focus on NSCLC, by integrating clinical regimens, resistance mechanisms, TME biology, and biomarker-guided translation across targeted and immunotherapeutic modalities.

## 2 Immunotherapy by the anti-cancer ICI or blocking drugs

Checkpoint blockade is designed to release inhibitory “brakes” on anti-tumor T cells, thereby restoring immune-mediated tumor control. In practice, the best-characterized clinical targets are PD-1/PD-L1 and CTLA-4, and antibodies against these pathways form the backbone of ICI therapy (10, 11). Rather than reiterating general definitions throughout this review, we refer to the introductory summary above and focus here on how these checkpoints are leveraged in therapeutic strategies and combinations relevant to lung cancer.

**Figure 1:**
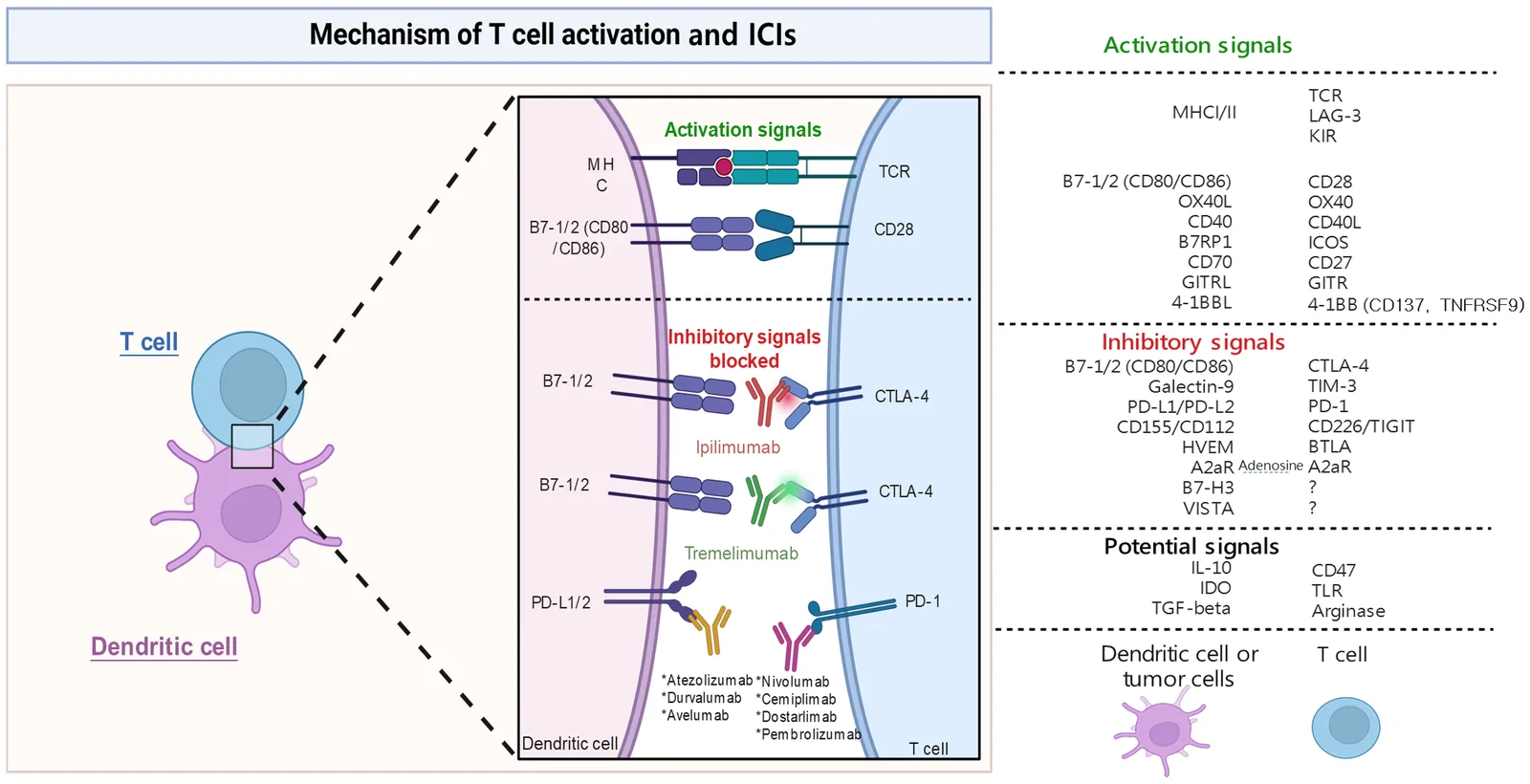
Various inhibitory and stimulatory immune checkpoints. Action mechanistic approaches of PD1/PD-L1– and CTLA-4–specific mAb immune checkpoints. Dendritic cells (DCs) express MHC, B7, or PD-L1, while T cells express CD28, CTLA4, or PD-1 on their surface. Binding and blocking of CTLA-4 by anti-CTLA-4 mAbs such as Ipilimumab or Tremelimumab induces inhibitory signaling of T cell’s cytotoxicity. 4-1BB and 4-1BB ligand: inducible costimulatory molecules. 4-1BB [CD137 or TNF receptor superfamily member 9 (TNFRSF9)]. 4-1BB is expressed in mouse B/T cells, DC cells, macrophages and NK cells, while 4-1BB is expressed in the B cells, activated CD4+ T and CD8+ T cells, DCs, monocytes, and DNK cells.

CTLA-4 is expressed on activated and regulatory T cells and competes with CD28 for binding to CD80/CD86 on antigen-presenting cells, thereby dampening early T-cell priming (12) (**Figure 1**). Ipilimumab was the first anti-CTLA-4 antibody to demonstrate an overall survival benefit in melanoma, establishing proof of concept for checkpoint blockade (13, 14). Subsequently, PD-1/PD-L1–directed antibodies expanded the clinical footprint of ICIs and enabled combination regimens (including PD-1 plus CTLA-4 blockade) in multiple settings (15, 16). Importantly, while these foundational observations were first made outside lung cancer, they provide the mechanistic basis for the lung cancer regimens discussed in later sections and tables. Key FDA-approved agents and indications are summarized in **Table 1**, and representative clinical trials are listed in **Table 2**.

**Table 2:** Clinical trials of PD-1, PD-L1, and CTLA-4–specific IC antibodies in cancer.

CT ID	Sponsor	Condition	Drug+combination	Phase
NCT04620200	Netherland Cancer Institute	SCC	Nivolumab or Nivolumab+Ipilimumab	2
NCT04154943	Regerneron Pharmaceuticals Partner: Sanofi	CSCC	Cemiplimab	2
NCT04710498	Stanford University, Partner: Genentech	Advanced CSCC	Atezolizumab	2
NCT03969004	Regerneron Pharmaceuticals (+Sanofi)	CSCC	Cemiplimab	3
NCT03834233	Instituto do Cancer do Estado de São Paulo	Advanced CSCC	Nivolumab	2
NCT05025813	Queensland Health (+Merck Sharp, Dohme Corp.)	CSCC	Pembrolizumab	2
NCT02760498	Regeneron Pharmaceuticals	CSCC	Nivolumab	2
NCT04242173	Regeneron Pharmaceuticals (+the H. Lee Moffitt Tumor Centre and Research Institute)	Malignant CSCC	Cemiplimab	2
NCT04050436	Replimune Inc. (+Regeneron Pharmaceuticals)	Malignant CSCC	Biological RP1+Cemiplimab	2
NCT04632433	Fondazione Melanoma Onlus	Skin SCC	Cemiplimab	2
NCT03684785	Exicure Inc.	MCC, CSCC, Melanoma, HNSCC, Solid Tumors	Cavrotolimod, Pembrolizumab, Cemiplimab	2
NCT05110781	Arnaud Bewley (+NCI, Genentech Inc.)	HNCSCC, HNSCC, Stage III CSCC of Neck AJCC	Atezolizumab	2
NCT04204837	Salzburger Landeskliniken (+BMS)	SCC	Nivolumab	2
NCT04808999	Diwakar Davar (+Merck Sharp, Dohme Corp.)	SCC	Pembrolizumab	2
NCT03944941	NCI (+Alliance for clinical trials in oncology)	Skin cancer	Avelumab, Cetuximab	2
NCT03284424	Merck Sharp & Dohme Corp.	SCC	Pembrolizumab	2
NCT03737721	EMD Serono, Alberta Cancer Foundation (+AHS Cancer control alberta)	Skin SCC	Avelumab	2
NCT01129154	Dr. Joseph Kerger, Partner: the Universitaires UCL de Mont-Godinne, Dr Lionel Duck, Cliniques Saint-Pierre Ottignies Clinics universitaires, Saint-Luc-Université Catholique de Louvain	SCC	Panitumumab	2
NCT03833167	Merck Sharp, Dohme Corp.	SCC	Pembrolizumab	3
NCT01979211	University of Cincinnat	SCC	Cetuximab	2
NCT02964559	Emory University (+Merck Sharp & Dohme Corp.)	Recurrent SCC	Pembrolizumab	2
NCT03836105	Regeneron Pharmaceuticals	BCC, CSCC	Cemiplimab	2
NCT02978625	NCI	MCC, Skin SCC	Nivolumab, Talimogene, Laherparepvec	2
NCT00240682	France Chartres Hospital France	SCC	Cetuximab	2
NCT03082534	Merck Sharp & Dohme Corp., (+Assuntina G. Sacco, MD)	CSCC, HNSCC	Pembrolizumab, Cetuximab	2

SCC, squamous cell carcinoma; CSCC, cutaneous SCC; MCC, Merkel cell carcinoma; NCI, National Cancer Institute; BMS, Bristol-Myers Squibb.

Although ICIs can generate durable responses, their use is constrained by immune-related adverse events, cost, and heterogeneous efficacy across patients. These limitations have accelerated combination approaches that aim to increase immunogenicity and remodel the TME, including partnerships with chemotherapy, radiotherapy, targeted therapies, and selected immunomodulatory agents (17, 18). The goal is to improve both response rates and durability without unacceptably increasing toxicity.

## 3 Key oncogenic driver, EGFR, and its gene mutation-specific TKIs in NSCLC

**Figure 2:**
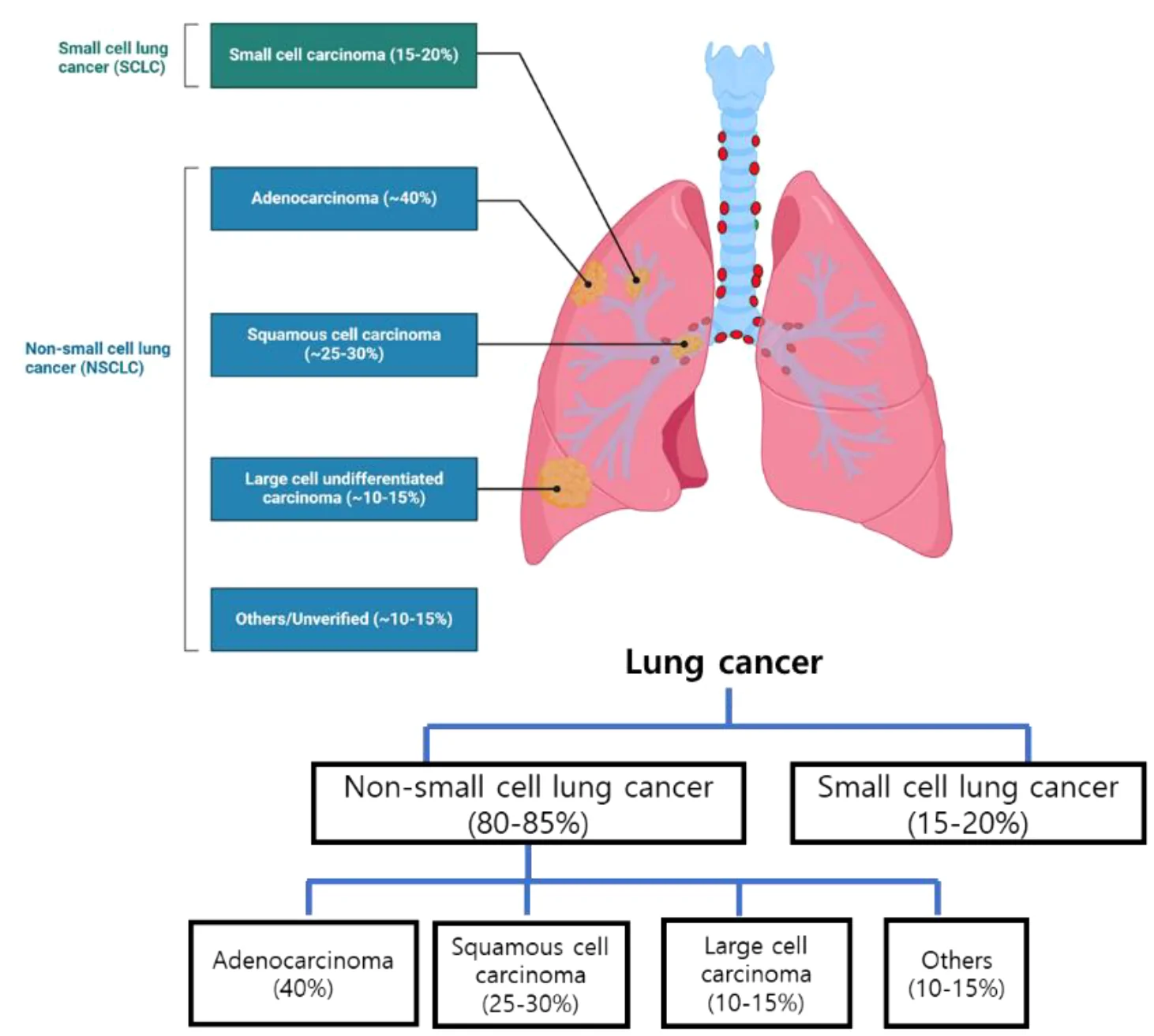
Classification of lung cancer.

**Figure 3:**
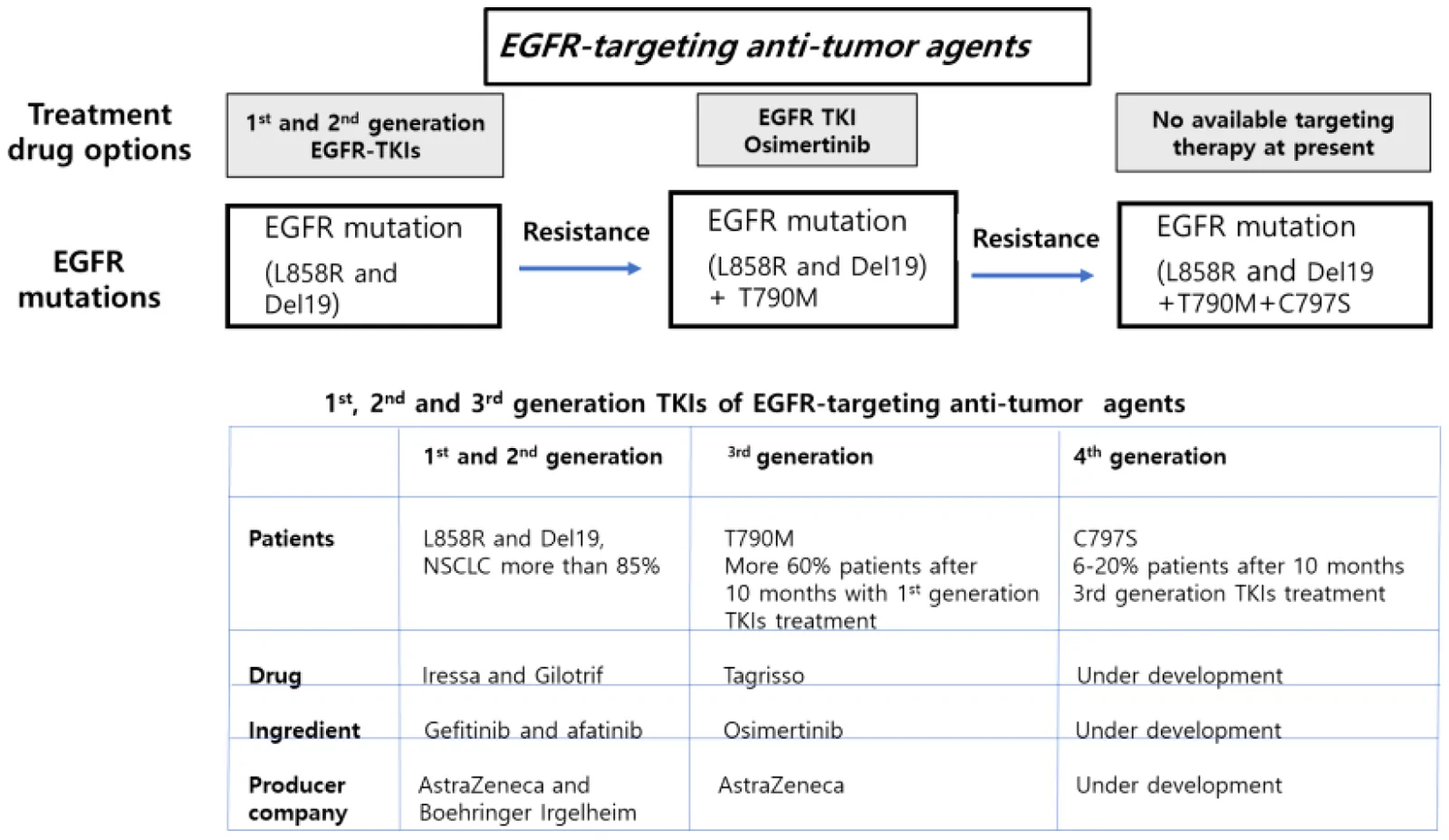
EGFR-targeted antitumor agents in lung cancer.

Lung cancer has largely been classified into the two major groups of NSCLC and SCLC (**Figure 2**). Lung cancer, as bronchogenic carcinoma, develops in the bronchi, bronchioles, and alveoli of the respiratory epithelia. Lung cancer has been known as a leading cancer death worldwide, as in the USA, lung cancer is a greater cause of death than colon cancer, prostate cancer, and stomach cancer. Epidemic growth factor (EGF), as a target for treatment, is abnormally activated in cancers, including NSCLC. Therefore, several anti-tumor agents targeting EGFR have been developed mainly in lung cancer (**Figure 3**).

EGFR-TKIs target EGFR, and a classical Gefitinib is effective for lung adenocarcinoma carrying sensitizing EGFR mutations. EGFR-TKIs drugs inhibit EGFR tyrosine kinase activity and are currently the preferred regimen for NSCLC. EGFR mutations (EGFRmut) in NSCLC are common. General EGFR mutations in NSCLC are sensitive to EGFR TKIs, but incurable EGFRs are not recognized by EGFR TKIs. These issues are an affordable resistance mechanism in EGFRmut. Micro-RNA–related mechanisms are not fully explained to support the resistance mechanisms. As the known first-generation inhibitors, reversible EGFR-TKIs are Erlotinib, Gefitinib, and Icotinib (19). Irreversible second-generation agents, including Afatinib and Neratinib, have been developed. Third-generation agents include Olmutinib and Osimertinib (20). Compared to conventional platinum-based chemotherapeutic agents, these agents have low cytotoxicity and high targeting advantages. They also show a high objective remission rate (ORR) and progression-free survival (PFS). Currently, resistance issue against EGFR-TKIs causes therapeutic difficulty in cancer patients within one year, although initial treatment with EGFR-TKIs to NSCLC patients yields a high efficacy of up to 80% (21). New EGFR-TKI drug classes, such as Mobocertinib and Tepotinib, also caused resistance within one year. Therefore, the resistant mechanism affordable for EGFR-TKIs must be elucidated. One EGFR-TKI resistant mechanism is the so-called drug efflux by drug transport proteins. Others are transcription factors in drug-resistant cancer cells that cause resistance via epithelial transformation. Potentially, autophagosomes in tumor cells degrade the EGFR-TKIs, and non-coding microRNAs may confer tumor cell resistance to EGFR TKIs.

### 3.1 Mutation issue in EGFR gene and EGFR mutant TKIs in NSCLC

Mutations in the EGFR gene are frequently observed and are particularly prevalent in adenocarcinoma patients from Asian populations of non-smokers. EGFR mutant forms promote cancer cell division. Caucasian NSCLC patients show a 15% EGFR gene mutation, and Asian NSCLC patients show a 30%–50% EGFR mutation. Asian female non-smokers and adenocarcinoma patients have higher EGFR mutation rates than others. EGFR^mut^ in NSCLC represents a cause of unsatisfactory life threat to patients (22). Commercially developed EGFR TKIs are curable for regular EGFR mutations found in NSCLC over 85% in cure. However, more than 15% of NSCLC EGFR mutations are not curable by regular EGFR TKIs due to TKI-resistant NSCLC EGFR^mut^. To increase the therapeutic outcomes of TKIs, the combination therapies with immunotherapy have been applied but exhibit EGFR TKIs resistance (23). To meet the medical demands, many inhibitors specific for different EGFR forms have been developed and approved as targeted anticancer agents. In addition, EGFR cDNA variation is associated with OS and PFS (24, 25). Therefore, EGFR TKIs are the current standard of care for NSCLC patients with EGFR mutations. Initially, several EGFR-associated TKIs are approved for first-line treatment in EGFR-bearing NSCLC. EGFR-bearing cancer cells, including NSCLCs, evolved to acquire the mutations of the EGFR genes. Treatment with TKIs prolonged the patient’s OS and PFS rates for EGFR mutation-bearing and malignant NSCLC. For example, treatment with TKI osimertinib can inhibit AURKA, AXL, Bcl-2, CDK4/6, JAK, MET, and PARP in patients with EGFR mutation-bearing and malignant NSCLC. In addition, the TKI combination with anti-VEGFR antibodies reverses the resistance to Osimertinib in cancer. ctcDNA can be resistant to TKIs. Due to the lack of drug targets in EGFR^mut^, chemo-immunological drug combination has been applied (26, 27) (**Table 3**).

**Table 3:** Current clinical trials for EGFR^mut^ forms.

Phase	EGFR-specific TKI name	EGFR mutation specificity	Specific agents
II	Not indicated	All EGFR^mut^	Atezolizumab, Bevacizumab, Carboplatin, Pemetrexed
Observation	Dacomitinib	T790M	Osimertinib
Observation	1st and 2nd generation TKIs	EGFR^mut^, TKI resistant (T790 negative)	Anlotinib, JS001
II	Not indicated	EGFR^mut^, ALK rearrangement, ROS1^mut^	Atezolizumab, (+/- Bevacizumab), Pemetrexed, Platinum
I	Not indicated	EGFR^mut/wt^	SPH1188-11
I	Not indicated	All EGFR^mut^	Necitumumab, Osimertinib
III	Not indicated	All EGFR^mut^	Trial A: Nivolumab, Platinum doublet chemotherapy, Trial B: Ipilimumab, Nivolumab Trial C: Platinum doublet chemotherapy
I	Not indicated	L858R, exon19del (mandatory) combination	CB-839HCl, Osimertinib
II	Not indicated	L858R or exon19del (mandatory)	+/- Bevacizumab, Osimertinib
II	Not indicated	EGFR exon20 mutation, HER2 exon20 mutation	Poziotinib
I-II	Osimertinib	L858R or exon19del	Necitumumab, Osimertinib, Trastuzumab
I	Osimertinib	L858R or exon19del	Alisertib (Sapanisertib), Osimertinib
I	Not indicated	EGFR^mut^ all exon19, exon21	MRX-2843, Osimertinib
I-II	Osimertinib	EGFR^mut^	GPX-001,Osimertinib
I	Osimertinib	L858R, exon19del	Osimertinib, Sapanisertinib
I	Not indicated	L858R, exon19 deletion, G719X, L861Q	Navitoclax, Osimertinib
III	Not indicated	EGFR^mut^ sensitized (exon20ins excluded)	+/- Bevacizumab, Osimertinib

### 3.2 EGFR exon 20 insertion (ex20ins) and EGFR (G719X, S768I, and L861Q)-targeting TKIs in NSCLC

For EGFR mutant TKI inhibitors, three classes have been developed for EGFR TKI, EGFR exon 20 insertion (ex20ins) TKI, and EGFR (G719X, S768I, and L861Q) TKI. General EGFR TKI inhibitors include Gefitinib, Osimertinib, Dacomitinib, and Lazertinib. Notably, an orally active and selective EGFR TKI, Gefitinib, sensitizes some EGFR mutations, such as a NSCLC mutant with an exon 19 deletion or L858R point mutation, and this has been used as a first-line drug for the mutant treatment. An orally active third-generation drug, Osimertinib (known as Tagrisso^®^) EGFR-TKI is also the NSCLC first-line drug for mutant treatment. Adult-targeted therapeutic drug Osimertinib is designed for EGFR mutation–bearing NSCLC who are previously not treated. Osimertinib resistance in EGFR efficacy is explained by characteristic phenotypes with *EGFR* amplifications, EGFR lysosome degradation, exon mutations (Ex18mut named L718Q or Ex20mut named C797S and L792X). Osimertinib resistance is also EGFR independently explained by gene amplification (*MET* and MAPK/ERK), AKT/PI3K signalings or gene fusions (28). For personalized therapy, Osimertinib has been approved in NSCLC patients with distinct EGFR^mut^ (ex19del and L858R) for adjuvant chemotherapy by the FDA (29). As a second-generation EGFR TKI, a first-line drug, Dacomitinib, is also effective for EGFR-mutant NSCLC. Dacomitinib is known to irreversibly bind to EGFR subtypes such as EGFR/Her1, EGFR/Her2, and EGFR/Her4 to inhibit the EGFR TK activities. A third-generation EGFR-TKI, Lazertinib (LECLAZA^®^), is orally effective and brain-penetrant for the patient treatment with NSCLC. As an irreversible EGFR-TKI, it inhibits the T790M mutant EGFR and activates EGFR mutants such as Ex19del and L858R while sparing EGFR wild-type NSCLC. From the properties, in 2021, Lazertinib acquired the FDA approval for the therapeutic treatment of NSCLC patients with EGFR T790M mutant malignancy for whom EGFR-TKI therapy has not previously been treated.

Exon20ins inhibitors include Amivantamab and Mobocertinib. Amivantamab is known as a mAb that specifically recognizes the MET proto-oncogene (MET) and EGFR. In 2021, Amivantamab acquired the FDA approval for the treatment of NSCLC patients with EGFR exon20ins who received the platinum chemotherapy. Amivantamab blocks ligand binding to EGFR, ligand binding to MET, and EGFR dimerization. Amivantamab also enables antibody-dependent cytotoxicity (ADCC) and downregulates cell surface proteins via the receptor-mediated internalization and phagocytosis. Amivantamab is effective for NSCLC with Exon20ins EGFR mutations. Ex19del and L858R EGFR mutations in NSCLC patients are developed after Osimertinib injection. Amivantamab is singly used to patients or by a combination therapy with a third-generation TKI, Lazertinib. Amivantamab in combination with chemotherapy is under clinical investigation of phase III clinical trials for the EGFR mutant NSCLC patients with Exon20ins who are not previously treated with tumor therapy. NSCLCs with Exon19del and L858R mutation are treated with a combination therapy with Lazertinib (MARIPOSA). Also, these patients are combined with Lazertinib and chemotherapy for the Osimertinib (MARIPOSA-2). Recently, a first-in-class Mobocertinib EGFR TKI (known as EXKIVITY™) has been designed for the hEGFR exon 20 insertion (EGFRex20ins)-positive NSCLC. Mobocertinib was approved in the FDA. As an EGFR (G719X, S768I, and L861Q) TKI inhibitor, a second-generation Afatinib is an irreversible blocker as an ErbB-family target for the EGFRm plus NSCLC. Afatinib is active against EGFR unusual mutations with S768I/G719X/L861Q and against common mutations with Del19/L858R. Afatinib is well tolerated.

### 3.3 Emerging TKI therapy–resistant EGFR mutations and unsatisfactory efficacy in NSCLC treatment

EGFR TKIs are still unsatisfactory in their efficacy in certain tumors due to TKI therapy–resistant EGFR mutations. Drug efflux pump or drug transporter proteins are aberrantly expressed in most drug-resistant cancer cells, indicating tumor drug resistance. Drug efflux pump or drug transporter protein genes encode the ATP-binding cassette (ABC) superfamily and solute carrier (SLC). ABC transporter proteins, designated ABC-A to ABC-G, are present in a large superfamily consisting of 48 members with 7 subfamilies. The representative ABC transporter proteins are classified into the multidrug resistance (MDR) P-glycoprotein (P-gp) group, and are named MDR1 (or ABCB1), ABCC2 (or MDR-associated protein MRP2) and breast cancer resistance protein (BCRP or MXR, ABCP2, ABCG2 or abcc2) (30, 31). Like P-gp, BCRP (ABCG2) belongs to the ABCG family and is expressed in MDR breast cancer cells (BCC). These proteins efflux xenobiotic drugs and function as MDR-associate protein-1 in the NSCLC drug resistance event. Thus, next generation TKI as allosteric inhibitor types have been considered for better efficacy against EGFR mutant forms that are conventional drug therapy-resistant, and these have been combined for effective therapy in NSCLC. Allosteric (ATP non-competitive) EGFR inhibitors have been developed to recognize a separate EGFR binding site from the previously recognized and existing site-specific TKIs for the ATP-competitive EGFR site (32).

## 4 Combination of the next-generation allosteric inhibitors against the EGFR inhibitor–resistant in NSCLC

Next-generation allosteric inhibitors have been designed for the earlier conventional therapy-resistant mutant forms of EGFR. These forms have been combined for effective therapy in NSCLC. To improve drug efficacy and overcome earlier conventional therapy-resistant NSCLC patients with genetic EGFR gene mutations, a strategy has been designed to develop allosteric (ATP non-competitive) EGFR inhibitors that specifically recognize a different existing EGFR site distinct from the previously known EGFR amino acid region for ATP-competitive TKIs of EGFR (32). Current fourth-generation EGFR inhibitors in clinical trials are all ATP-competitive inhibitors. To overcome drug resistance mutations, combination therapy with Tagrisso can maximize the treatment’s efficacy. This enhances the efficacy and overcomes the drawback of the previous Tagrisso. EGFR mutations of L858R, T790M, or C797S confer available drug resistance, which is considered the undruggable mutation of the EGFR gene. In fact, the Osimertinib resistance was known in the case caused by the EGFR mutation C797S. If a specific mutant clinically impedes the formation of a covalent bond with irreversible inhibitors of EGFR kinase, such inhibitors will be desired. The C797S-mediated drug resistance is still to be overcome. EGFR-L858R, T790M, or C797S mutation-targeting EGFR inhibitors are being developed for NSCLC patients. Combination regimens with Tagrisso (or Osimertinib), resistant C797S-targeting first and third generation TKIs of EGFR are being used for patients with NSCLC mutants (33).

L858R mutants are characteristic of EGFR-sensitizing, exon-19’s del19, and exon-21’s L858R point mutations, accounting for 85%–90% in total. Among them, exon-19 mutants show a high drug sensitivity. EGFR T790M acquired resistance mutations occur in 60% of patients treated with first generation EGFR inhibitors for 10 months. Then, to improve the resistance acquisition issue of first generation EGFR TKIs, second generation TKIs of EGFR have been developed, such as Giotrif (or Afatinib) that irreversibly blocks all ErbB family signaling. However, the second generation EGFR inhibitor Giotrif showed a low resistance-overcoming effect on the pre-existing inhibitors and an adverse cytotoxic effect to kill normal cells. Thereafter, to improve the resistance acquisition issue against first generation TKIs of EGFR, third generation TKIs of EGFR, as in Tagrisso, have been developed. Tagrisso acquired the FDA approval in December 2015 for metastatic EGFR-mutant patients with T790M mutated NSCLC. Additionally, because Tagrisso irreversibly binds to several EGFR mutants, including T790M, L858R, and exon19 deletion, Tagrisso acquired the FDA approval for a first-line option of therapy for metastatic EGFR mutant NSCLC patients in 2018. The EGFR C797S mutant has been known to bear an EGFR-acquired resistance mutation. When clinical patients with NSCLC are treated with third-generation EGFR TKIs, a covalent bond-specific C797S acquired resistance mutation occurs after 10 months of drug treatment. Drug-resistant acquired EGFR mutations have been reported during first- and second-line treatment with Tagrisso. For example, C797S acquired resistance mutations have been reported in 6%–10% of first-line Tagrisso patients (dual point mutation at L858R or del19/C797S) and 10%–20% of second-line Tagrisso patients (triple point mutation at L858R or del19/T790M/M/C797S) (34). T790M instability is known. After the approval of the first-line drug Tagrisso, the rate of the dual point mutation rather than the triple mutation has decreased.

More than 85% of cancer patients with NSCLC have EGFR mutations. Among them, 30%–40%, L858R 40%, and del19 50% are GFR L858R mutants: 9%–67% (USA) and 40%–60% (Asian) are Tagrisso resistant mutation C797S (35). The market of a third-generation EGFR TKI Tagrisso is 3.4 billion $ in 2022 and is currently used as a first-line treatment option in 80 countries, and also global sales of Tagrisso are gradually increasing (2021–2031 compound annual growth rate 2.6%). (1) Overcoming a third-generation EGFR TKI Tagrisso demerit and resistance is required: Tagrisso and EGFR TKI are not effective for L858R patients. Tagrisso-resistant mutant (C797S)–targeting inhibitors are still not developed. T790M and C797S ATP competitive inhibitors show skin toxicity and cardiotoxicity as adverse effects to be overcome. For the heterogeneity of various mutational combinations, a combination treatment strategy is required. Resistance-improved EGFR TKI is required. (2) For the EGFR L858R mutation and resistant occurrence rate (L858R mutant occupies 40% and del19 for 50% rate), regarding the incidence of resistance mutations of EGFR L858R, EGFR mutations have L858R 40% and del19 50%. The first-generation EGFR TKI Iressa (or Gefitinib) or Tarceva (or erlotinib) during their treatments causes each drug resistance, where T790M has about 50%–60%. Iressa (or Gefitinib)-resistant mutant T790M occupies about 50%–60% rate. Tagrisso-resistant mutant C797S occupies about 22% (occurrence of 7% in the first treatment and 15% in second treatment), based on the phase 3 FLAURA clinical trials. Depending on the increasing market size of Tagrisso, the fourth generation EGFR inhibitor market is also expected. Combination treatment with Yagrisso is desired. Rybrevant (Amivantamab), a dual EGFR/cMET-targeting Mab that was approved by the FDA in 2021, has overcome combination therapeutic drug has been needed. Although combination treatment with Lazertinib can target lung cancer C797S patients, a combination of Lazertinib and ATP competitive inhibitors can still lead to Lazertinib-resistance issues and cause interstitial lung disease to be overcome (36). For additional EGFR mutant patients, Lazertinib, as a third-generation TKI of EGFR, has been used as an additional first-line option of therapy for EGFR mutant NSCLC patients who bear either an exon21 L858R mutation or an exon19 deletion.

For future strategy, Tagrisso and first-line EGFR L858R TKI as a combination treatment option, and C797S-targeting EGFR TKI as a single treatment option are required to be developed for lung cancer therapy. In addition, allosteric (ATP non-competitive) EGFR inhibitors are required to develop. Tagrisso-resistance-overcoming fourth-generation EGFR TKI (first in class) that overcomes ATP-competitive EGFR TKI’s demerits of resistance mutation. EGFR-targeting low molecules can be synthesized with a defined structure basis and a simulated dynamic approach. This is also another evolved generation of EGFR-targeting anti-tumor agents in lung cancers, as science is future-forwarded (**Table 4**) (37).

**Table 4:** Evolved generation of EGFR-targeting anti-tumor agents in lung cancers.

1^st^, 2^nd^ and 3^rd^ generation TKIs of EGFR-targeting anti-tumor agents			
	1^st^ generation and 2^nd^ generation	3^rd^ generation	4^th^ generation
Target patients	L858R and Del19, 85% more of NSCLC patients	T790M More 60% patients after 10 months 1st generation TKIs treatment.	C797S 6-20% patients after 10 months 3rd generation TKIs treatment
Product name	Iressa and Gilotrif	Tagrisso	Under development
Ingredient name	Gefitinib and afatinib	Osimertinib	Under development
Development company	AstraZeneca and Boehringer Irgelheim	AstraZeneca	Under development

### 4.1 Critical synthesis on question why combination of RTK/EGFR inhibitors and ICIs fail or succeed in lung cancer

Based on a catalog of approvals, a question is raised why some combination regimens deliver durable benefit in lung cancer, producing limited efficacy or unacceptable toxicity. Generally, combinations increase tumor antigen release and immune priming, enhance T-cell trafficking and retention within the TME, and alleviate dominant immunosuppressive circuits including myeloid- or stromal-driven exclusion) without severe tissue inflammatory response. RTK pathway inhibition like EGFR-targeted therapy can synergize with IC inhibition by reducing oncogene-mediated immune evasion. However, in oncogenic-driver NSCLCs such as EGFR- or ALK-based tumors, clinical responses to PD-1/PD-L1 blockade are often modest and characterized by low baseline T-cell infiltration and distinct myeloid populations. Therefore, simply ICIs-combined RTK inhibitors do not induce such desired immune responses if the dominant barrier is immune exclusion rather than IC-mediated exhaustion. In addition, several RTK inhibitor–combined ICIs show their limitation caused by toxic issues. Mechanistically, RTK inhibitors increase epithelial and endothelial stress responses, while ICIs amplify immune stimulation, predisposing to toxic inflammatory responses that constrain dosing and compromise treatment. Such observed arguments are fit for therapeutic designs that separate action goals (tumor regressing vs. immunological-primed effects) through biomarker-specific targeting.

Allosteric RTK inhibitors improve the therapeutic efficacy of RTK–ICI combinations. Non-ATP site or allosteric regulatory pocket-based agents can selectively enhance mutants with off-target kinase inhibition. In addition, allosteric inhibitory agents can suppress adaptive signaling during combination with Orthosteric inhibitors (e.g., Osimertinib) or signaling modulators, lowering dosing. From an immunologic standpoint, selective and controllable signaling suppression is desired for combinatory approaches with ICIs due to their oncogene-based immunosuppression and their lowered toxicities. In fact, RTK-ICI combination in lung cancer is synergistic when co-mutations, pathway activation, immune biomarkers such as PD-L1, TMB, and T-cell infiltration, and interferon signaling are incorporated. Under this issue, by improving pathway control and tolerability, allosteric RTK inhibitors may facilitate effective checkpoint blockade in targeted lung cancers.

## 5 Mutation-specific inhibitors in various cancers

### 5.1 ALK mutation and inhibitors in NSCLC

ALK mutation occupies 2%–5% of adenocarcinoma patients of NSCLC. The ALK mutation is frequently detected in non-smoker, young people and women. The ALK mutation includes rearrangement, amplification and point mutation (38). The ALK therapeutics include Crizotinib (first generation), Alectinib and Brigatinib (second generation), and Lorlatinib (third generation) as non-payment item at the market. Crizotinib TKI is used for the therapeutic agent of ALK or ROS1-bearing NSCLC patients, and also for ALK-bearing anaplastic large cell lymphoma patients and inflammatory myofibroblastic tumor patients (39). Crizotinib was the FDA-approved drug in 2011 for ALK and ROS1 rearrangements. Crizotinib targets the fusion protein of echinoderm microtubule-associated protein-like (EML)-4 and ALK, EML4-ALK, of NSCLC patients. Crizotinib was the first-in-class drug for ALK-positive tumors. Second- and third-generation ALK-TKIs overcome Crizotinib. Ceritinib is a first-generation ALK inhibitor that is treated for adult’s NSCLC patients with ALK-bearing malignant tumors following therapy-failed outcomes caused by secondary resistance or intolerance of prior Crizotinib therapy (40). Limited population number with 4% of NSCLC patients are known to bear a chromosomal gene fusion rearrangement between EML4 and ALK, which constitutively generates kinase activity that causes carcinogenesis and the malignant phenotype changes. Ceritinib inhibits ALK autophosphorylation, downstream signaling protein STAT3 phosphorylation mediated by ALK, and ALK-dependent growth of cancer cells. When the tumor cells are treated with Crizotinib, the tumors show the drug resistance caused by the enzyme gene mutations in the amino acid residues known as “gatekeeper”. To overcome and improve the mutation-based drug resistance issue, so-called second-generation inhibitors of ALK have been designated and developed. Representative drugs such as Ceritinib can overcome drug resistance against Crizotinib. In April 2014, Ceritinib has been FDA-approved with an extremely high response rate (RR) against tumors with Crizotinib resistance with 56% rate and has been designated in an orphan drug level.

### 5.2 ROS1 and RTK mutation and inhibitors in NSCLC

The ROS1 mutation type is rare compared to the ALK mutation. The ROS1 gene mutation shows about 1%–2% of lung cancer patients, mainly in adenocarcinoma NSCLC (38). There are two ROS mutation inhibitors, Crizotinib and Entrectinib. ROS1-positive patients are younger than the lung cancer patients in non-smokers or light smokers. ROS1-targeted inhibitors include the pain medication (Crizotinib) and non-reimbursed drugs of selective inhibitors of tyrosine receptor kinase (TRK) fusion protein that also act on the central nervous system (CNS). TRK genes join or “fuse” with an unrelated gene and generate a TRK fusion protein. The TRK fusion protein is highly active in causing a tumor phenotype to proliferate the cells. An inhibitory drug, Entrectinib, is a pan-inhibitor with ALK, ROS1, and TrkA/B/C inhibitory potentials (41). Entrectinib also induces autophagy. Entrectinib selectively inhibits the neurotrophic T receptor kinase (NTRK) and ROS1. Therefore, Entrectinib is used to treat solid tumors harboring NTRK gene fusions and NSCLC patients with ROS1 mutations.

### 5.3 BRAF mutation and inhibitors in NSCLC

After EGFR activation, BRAF functions as a key signal transduction regulator, and BRAF mutation induces uncontrolled signaling related to growth and regulation. Among NSCLC patients, 2% of patients have BRAF mutations, and the frequency rate is higher in smokers of adenocarcinoma patients than in non-smokers (42). Compared to other mutations, OS and PFS lengths are relatively short. BRAF mutation inhibitors are Dabrafenib (Rafinlar® for Novartis and Tafinla® for GSK 2118436A) and Trametinib (43).

### 5.4 NTRK mutation and inhibitors in NSCLC

NTRK is a brain-derived neurotrophic Trk (Tropomyosin-related tyrosine kinase) and consists of NTRK1, NTRK2, and NTRK3 (44). NSCLC patients account for 1%, and other information is not available. Recently, NTRK gene fusion lung cancers have been regressed by Bayer’s Vitrakvi^®^ (Larotrectinib) with long-term response and safety. Entrectinib is also an NTRK inhibitor (45).

### 5.5 Exon-14 skipping mutation-bearing mesenchymal epithelial transition gene and inhibitors in NSCLC

MET gene exon-14 (METex14)-skipping mutations are found at a low frequency, ranging from 3.0% to 4.0% of NSCLC (46), mainly in elderly NSCLC patients. Primarily, MET-targeting inhibitors are effective but show their tolerance to NSCLC patients with the skipping mutations of METex14. Furthermore, chemotherapy and immunotherapy are not fully proven for efficacy and safety. In a non-randomized phase 2 study by an open-labeled Tepotinib, developed as a MET inhibitor, exhibited 18 months of median duration of response (DOR) in METex14 mutant advanced/metastatic NSCLC patients, with a highly valued 51.4% positive response rate. Then, MET inhibitor Tepotinib acquired the FDA approval for the malignant NSCLC METex14 skipping mutation-carrying patients as a treatment option (47). However, MET inhibitor Tepotinib has been approved by the European Medicines Agency (EMA) only for the treatment of NSCLC patients who received the previous treatment with chemotherapy by platinum-based drugs and/or immunotherapies (48). METex14-mutated malignant NSCLC with PD-L1 expression is also capable of treatment with a first-line Tepotinib. However, immunotherapy with or without chemotherapy is the first-line therapeutic approach of the standard therapeutic option for metastatic NSCLC patients without alteration of driver molecules (49). More specifically, single anti-PD-L1 antibody immunotherapy is the treatment option for NSCLC patients with high PD-L1 expression. PD-1-specific Pembrolizumab antibody treatment as a single and first-line therapy exhibited a higher median OS value than chemotherapy with platinum-based drugs for the treatment of metastatic NSCLC patients in the phase III KEYNOTE-024 trial.

Current treatment options for METex14 mutation-related metastatic NSCLC are limited. Moreover, the combined immunotherapy as the first-line option of platinum-based chemotherapy showed the increased survival level regardless of PD-L1 expression over single chemotherapy treatment (50). In addition, the optimized treatment is not conditioned yet for NSCLC patients with skipping mutations of METex14. Currently, randomized studies on the efficacy of chemotherapy and/or immunotherapy are not available in the METex14 mutant NSCLC patients because of an absence of summarized data. Immunotherapy for these NSCLC patients with METex14 skipping mutations is not distinctive regardless of PD-L1 expression (51, 52). In the near future, randomized comparisons are required for immunotherapy with MET inhibitors and chemotherapy, although no current outcomes are available for METex14-mutant metastatic NSCLC patients (53).

Clinical and selective MET inhibitors to METexon14-mutated NSCLC also include Tepotinib, Capmatinib (TABRECTA^®^, Novartis, Basel, Switzerland) and Savolitinib. MET mutation is required for growth, survival and metastatic potentials of tumor cells. 2% of lung cancer patients have MET amplification. MET is highly related to EGFR and Met mutant T790M of EGFR is highly active in MET pathway (54). The FDA-approved MET inhibitor Capmatinib is effective for metastatic NSCLC therapy of adult patients with Met mutant T790M of EGFR, in MET exon 14 skipping. Similarly, tepotinib (EMD Serono Inc., Boston, Massachusetts, United States) is also approved as non-reimbursed drug (55). MET inhibitor TEPMETKO^®^ (Tepotinib) is effective for the therapeutic treatment of adult metastatic patients with skipping mutations of MET Exon 14 gene. Metastatic NSCLC patients with skipping mutation of MET exon 14 and surfaced expression of PD-L1 are mainly interested in receiving of the MET inhibitors such as Tepotinib. As a first-line therapeutic option, Tepotinib is effective for NSCLC patients with skipping MET exon 14 gene mutation, regardless of surfaced expression of PD-L1.

### 5.6 RET fusion mutation and inhibitors in NSCLC

RET (rearranged during transfection) fusion is an oncogenic driver in 1%–2% of NSCLC. In NSCLC patients with RET fusion, selective RET fusion inhibitors are not well summarized for the current aspects of safety and efficacy. RET alterations include fusions, point mutation activations, and acquired drug resistance mutations. A selective RET fusion inhibitor, Selpercatinib penetrates the CNS. Retevmo^®^ (Selpercatinib) (LOXO-292, ARRY-192) is a specific RET (c-RET) inhibitor as the first FDA-approved agent for advanced RET type NSCLC, thyroid cancers, and certain cancers. Selpercatinib as a small molecule inhibitor is an ATP-competitive RET kinase inhibitor specific to RET alterations (56). As a RET inhibitor, Selpercatinib is also known as Retevmo that affects both tumor cells and healthy cells, potentially causing unexpected and serious side effects.

### 5.7 EGFR-2 (HER2) mutation and its inhibitors in NSCLC, gastric and breast cancers

Human EGFR-2 (HER2) is frequently mutated in many tumor cells and HER2 mutation has been strategically targeted. The first approved medicine, ENHERTU (derived from the en-HER-too and approved by the FDA as Trastuzumab Deruxtecan), treats adult metastatic patients with HER2-bearing breast cancers. ENHERTU is a humanized mAb, trastuzumab conjugated with a topoisomerase I inhibitor, Deruxtecan. As such, ENHERTU is a representative antibody-drug conjugate (ADC) form and a mAb Trastuzumab-Deruxtecan covalently linked form. The licensed ADC, ENHERTU is effectively treated for human breast and gastric cancer patients as well as gastroesophageal adenocarcinoma patients. NSCLCs that have a HER2 mutation and stomach cancers with HER2 such as gastroesophageal junction and gastric adenocarcinoma are also treated by ENHERTU. Although ADC technology is popular in new drug application (NDA) throughout the world, biosimilar drug development is not always successful (57). For example, Korean company Hanmi Pharmaceutical Co. has tried to launch a biosimilar ADC, EGFR TKI Poziotinib, due to approval issues from the FDA (58).

### 5.8 Kirsten rat sarcoma mutation and inhibitors in NSCLC

Lung cancer has been frequently found in human patients and is thus classified to the world’s highest level of occupied cancers. In 2020, new lung cancer cases will exceed 2.21 million and annual lung cancer deaths will exceed 1.8 million. Lung cancer comprises two main types of small cell lung cancer (SCLC) and NSCLC. Most lung cancers (85%) belong to NSCLC and 15% of lung cancer is classified to SCLC cases. In lung cancer patients, KRAS determinants are used as molecular switch for on-and-off signaling pathways for cancer cell proliferation and also serve as an instructor for whether a cell goes through or stops its proliferation in regular states of growth. KRAS mutation is mostly important for the targeted agents against lung solid cancers. Enhancement of response rate of KRAS mutation–derived lung solid cancers and combination therapy are crucial for the cancer treatment. KRAS strongly binds to GTP with binding strength (Kd) at pM ranges (59). Thus, development of low molecular inhibitors that are competitive against the binding capacity is important. However, KRAS structure lacks allosteric inhibitor-binding pocket, suggesting a difficulty in its inhibitor designation. Moreover, poor prognosis cancers such as specific pancreatic ductal adenocarcinoma show diverse KRAS mutations. KRAS mutation enhances the GTP binding from GDP and thus development of specific inhibitors that covalently bind to the KRAS-GDP binding region is not easy.

## 6 KRAS and KRAS G12C inhibitors in NSCLC

### 6.1 Development of KRAS-targeted anticancer agent and KRAS G12C-covalent binding inhibitors in NSCLC

RAS mutations are broadly distributed across human cancers, and KRAS alterations are particularly prevalent in lung cancer, where they represent a major actionable driver in a clinically important subset of NSCLC. Although KRAS mutation frequencies are also high in other tumor types (e.g., pancreatic and colorectal cancers), we cite those data only to contextualize KRAS biology; in this review, we focus on how KRAS mutations shape immune phenotypes, therapeutic resistance, and ICI-combination opportunities in lung cancer. Approximately 85% of RAS-driven cancers harbor KRAS mutations. Historically, RAS inhibitors have been investigated for decades, yet RAS was long considered an “undruggable” target (60). In NSCLC, KRAS mutations occur in a substantial proportion of adenocarcinomas, motivating the development of direct KRAS inhibitors and rational combinations with ICIs.

#### 6.1.1 Roles of small GTPases of HRAS, NRAS and KRAS isoforms

RAS is a small GTPases protein crucial for signaling transduction process of cell differentiation, growth and survival and consists of HRAS, NRAS, and KRAS 3 isoforms. They as tumor genes show mutation in tumor cells. KRAS mutation is frequently associated with the continued cell growth in abnormal status. Moreover, KRAS mutation often triggers the most metastatic phenotypes of the subject cancers. Among them, a KRAS mutation form named KRAS G12C is most common in NSCLC with a strong aggressiveness, with a frequency rate of approximately 14% of NSCLC patients. Therefore, large efforts to develop KRAS-targeted anticancer agent and KRAS G12C–covalent binding inhibitors have been enforced in academia and industries. Upon successful development of KRAS G12C–covalent binding inhibitors, the combination therapeutic approaches have been generalized. Hopefully, there was a huge illegible reason for breakthrough of KRAS mutation-targeted inhibitor development because KRAS mutations show structural distinct of protein and thus inhibitor development has not been easy. However, in 2013, UCSF’s Dr Keven Shokat discovered a new pocket that G12C cysteine located on adjacent KRAS (G12C) protein Switch II, interacts with a covalent inhibitor (61). The discovered pocket is an allosteric pocket located on the low side of the KRAS beta-sheet during KRAS structural investigation on G12C mutation. They have also discovered that the GDP-bound KRAS only exposes this allosteric pocket. To bind to this allosteric pocket, they developed several low molecular compounds that covalently bind to Cys residue in the pocket by keeping the inactive KRAS form, KRAS-GDP form. This pocket has not been found in Apo crystal structure. However, this pocket is formed only when KRAS (G12C) protein is present in an inactive GDP-bound form and a very tiny allosteric binding pocket. This discovery of a pocket allows development of KRAS inhibitors.

#### 6.1.2 Development of GDP-bound KRAS specific inhibitors for KRAS G12C mutation with the KRAS-GDP state

KRAS-mutant subtype G12C inhibitors have successfully been developed using the discovered pocket. Because the KRAS G12C mutation relatively keeps the KRAS-GDP state, GDP-bound KRAS specific inhibitors can be created. However, the KRAS G12C mutation can also take KRAS-GTP form and also non-G12C mutations can show the KRAS-GTP forms. Therefore, it is so important to develop inhibitors that can bind even to the active KRAS-GTP form. Recently, Revolution Medicine CO. Ltd. tests the KRAS-on inhibitors that can bind to KRAS-GTP state in the non-clinical trials (62). Although combination therapeutic efficacy between KRAS-on inhibitors and PD-1 inhibitors (Keytruda) is not observed, a triple combination therapy of the KRAS G12C inhibitor+PD-1 inhibitor+SHP2 inhibitor showed the increased overall response rate (ORR) in the non-clinical trials (34th EORTC-NCI-AACR Symposium, Oct. 26-28, 2022, Barcelona, Spain).

In 2018, Amgen started a large pre-commercial test via clinical trials of first-in-class KRAS (G12C) inhibitor Sotorasib and FDA approved in 2021 for treatment drug of KRAS (G12C) mutation-bearing NSCLC patients who are locally metastatic or advanced and not received for previous therapeutic treatments (63). Recently approved KRAS inhibitors, Sotorasib (2022.5.18. FDA, Amgen’s targeted anticancer agent Lumakras, Sotorasib) and later, Krazati (KRAZATI^®^, Adagrasib, Mirati Therapeutics, FDA, 2022. 12) target mutant KRAS p.G12C that occupies 33% of the mutant KRAS. Recently, combination therapeutic strategies have clinically been examined to overcome short drug reaction period. Thereafter, Mirati Therapeutics has also clinically been studying additional KRAS (G12C) inhibitor Adagrasib and FDA in 2022 approved Adagrasib for KRAS (G12C) mutation-bearing NSCLC patients who are locally advanced or metastatic (64). Adagrasib can clinically penetrate into CNS for CNS-invaded NSCLC patients as a merit point. Combination therapeutic strategies of Adagrasib are also examined to overcome short drug reaction period. Except for the KRAS mutant subtype p.G12C, the remaining 66% of G12X type is actively under development. p.G12C KRAS mutations are also found in 1%–2% of pancreatic cancers. Amgen has succeeded to develop Sotorasib (LUMAKRAS^®^) that targets KRAS G12C mutation in 2021. LUMAKRAS treatment is effective for the metastatic patients of KRAS G12C adult NSCLC, and it has been finally approved by FDA, based on DOR and ORR (65).

#### 6.1.3 Development of RAS GTPase family inhibitors

Continuously, in 2022, FDA approved the Mirati Therapeutics’s Adagrasib (KR KRAZATI^®^) as a second KRAS G12C inhibitor for NSCLC patients who harbor the KRAS G12C mutation. Moreover, FDA further approved a RAS GTPase family inhibitor, Adagrasib (Krazati, Mirati Therapeutics) via accelerated process on December 12, 2022, for adult NSCLC patients who have KRAS (G12C) mutation and local advance or metastasis. Separately, the therascreen KRAS RGQ tissue PCR kit by QIAGEN and a qualitative NGS-based Resolution plasma ctDx FIRST assay by Agilent have also been approved by FDA as companion diagnostics for Krazati. The therascreen KRAS RGQ PCR assay is qualitative *in-vitro* diagnostic detector of the KRAS oncogene mutation. It uses formalin-fixed and paraffin-embedded DNA extracts of NSCLC tissue. Companion ctDx FIRST assay detects KRAS G12C in NSCLC due to accurate diagnostic to KRAZATITM (Adagrasib) and profiles tumor mutations in detection of single nucleotide variants and EGFR deletions. Plasma specimen shows a mutation, no more tests are not required but when mutation is detected, the tumor tissue is examined. FDA-approved key point of KRYSTAL-1 is based on open-label clinical trial (NCT03785249) for metastatic or locally advanced patients with KRAS G12C mutation-harboring NSCLC (66). The therapy-received patients with KRAS G12C mutant NSCLC were the subjects with ICI or platinum-based chemotherapy under concurrent or sequential treatment. Doses of Adagrasib 600 mg were treated to patients twice daily by oral administration until unacceptable toxic status or disease progression. Adagrasib by clinical II trials of combination therapy with Keytruda (Pembrolizumab) showed 49% of tumor regression rate of patients, indicating superior activity rather than the previous sotorasib (67).

KRAS G12C inhibition drugs induce aggressive phenotype of tumor associated macrophage of cancer patients as reported by the non-clinical trials and clinical trials (57). Hence, immune anti-cancer agent, ICI anti PD-1 mAbs have been used for combination with the KRAS inhibitors in the clinical trials. Krazati is an irreversible KRAS G12C inhibitor with a highly selectivity and potent as a KRAS G12C-specific inhibitor. Chemically, Krazati binds to the mutant Cys amino acid residue in KRASG12C by a covalent bond. Krazati also prevents downstream signal transduction, not affecting the wild-type KRAS protein without any wild-type KRAS alteration because it structurally locks the inactive state of mutant KRAS protein. KRAZATI^®^ (Adagrasib)’s mechanism of action is distinctive for anti-cancer progression. KRAZATI^®^ (Adagrasib) prevents proliferation of tumor cells and blocks tumor cell viability in KRAS G12C-mutant tumor cells. It causes regression of KRAS G12C-mutated tumor cells in its graft xenotransplanted model animals without off-targeting activity (68).

#### 6.1.4 Combination treatment of KRAS G12C inhibitor and ICI in NSCLC

Mirati Therapeutics (Mirati)’s 2022 report evidenced the combination treatment of KRAS G12C inhibitor Adagrasib and Merck’s Keytruda to NSCLC patient first clinical trials. Patients’ objective response rate (ORR) (more than 30% reduction in tumor size) shows 49% with disease control rate of 100%, as excellent outcomes. This data is clearly contrasted to the previous outcomes that Amgen’s Sotorasib and Keytruda combination causes liver toxicity and ORR of 28%, leading to clinical failure. Presently, Adagrasib’s market potential has been highly evaluated compared to that of Sotorasib. A highly selective RAS GTPase inhibitor Krazati™ (Adagrasib) is a low molecular drug specific for the target therapy of adult NSCLC patients who harbor a local KRAS G12C mutation with metastatic advanced tumor types and have been previously treated with at least a systemic therapy. The first KRAS inhibitor approved in May 2021 is recorded for Amgen’s Lumakras (Sotorasib) (69). Therefore, the second KRAS inhibitor, Mirati’s Krazati™ (Adagrasib) approved by the FDA is targeted for previously treated and advanced NSCLC patients. Krazati has been immediately released in 200 mg dosage of oral administration. Currently, Krazati drug is under investigation for KRAS G12C mutant-bearing advanced solid cancer patients, including colorectal, NSCLC and pancreatic cancers in both monotherapy and combination therapy with other anti-cancer agents.

Additionally, it can be used as a companion diagnostic to identify the KRASG12C mutation. Agilent and Mirati have developed a liquid biopsy companion diagnostic test called Agilent Resolution ctDx FIRST. Also, Mirati has developed a tissue-based Therascreen^®^ KRAS RGQ PCR kit by collaboration with a German company QIAGEN. These two different diagnostics commonly identify cancer patients who harbor genetic mutations of KRASG12C. FDA has proved these two tests for the therapeutic application of Krazati of advanced NSCLC treatment in 2022 and the drug itself has been approved by FDA in an accelerating case. In parallel, the NDA has been accepted in February 2022 by FDA for Adagrasib NDA filing (69). Simultaneously, a marketing authorization application has been filed to the EMA by Mirati in May 2022 for the therapeutic treatment of Adagrasib for the NSCLC patients with KRAS G12C mutation who were previously treated.

Blinded independent review has determined the efficacy outcomes primarily measured for DOR and ORR by response evaluation criteria in solid tumors (RECIST v1.1). The resulting outcomes derived from the patient measures showed an ORR of 43% with 80% of disease control and a median DOR of 8.5 months. The commonly observed side effects appeared in the subject patients were appetite reduction, diarrhea, hepatotoxicity, dyspnea, musculoskeletal pain, nausea, edema, renal impairment, tiredness and vomiting in the clinical trial.

### 6.2 Action mechanism and issues of pre-existing KRAS inhibitors and combination therapy

Genetic alterations in cancer cells lead to resistance to treatment, leading to cancer relapse (recurrence). In addition, the response rate (RR) of KRAS G12C is not high in comparison to EGFR and ALK-targeted anticancer agents because there are some mechanistic differences in drug resistance. In *in vitro* cellular models, Sotorasib or Adagrasib can appropriately inhibit their target RAS pathway, but the levels of its biomarker pERK are recovered in a negative feedback mode, and also the RAS pathway is reactivated as time passes. Resistance of KRAS mutant tumors against KRAS (G12C) inhibitors has occurred. KRAS (G12C) inhibitor-insensitive secondary KRAS mutations have been found in solid tumor cells. In addition, other types of genetic mutations generate intrinsic and adaptive resistance to tumor cells (70). For example, KRAS (G12C) inhibitor Adagrasib resistant cancer patients showed secondary KRAS mutations and other genetic alterations, with repeated genetic alterations. Thereafter, the conceptual combination therapy of pan-KRAS inhibitors has been considered for KRAS inhibitor-SOS1 interaction.

In targeted cancer clinical trials, KRAS mutant patients have additional mutations that interfere with inhibitor binding to the target. In clinical trials of KRAS inhibitors, ctDNA (circulating tumor DNA) sequencing data show new drug resistance or recurrent RTK of patients, such as FGFR and EGFR mutations. Especially, KRAS on-target mutation, such as Y96D, has also occurred. Big pharma companies such as Revolution Medicine Co. Ltd. that develop KRAS-targeted anticancer agents simultaneously target SOS1 or tyrosine phosphatase-2 (SHP-2) to enhance the RR to overcome the limited KRAS inhibitors (71). The GDP-bound inactive SWI/SWII RAS form, which is in the “OFF” state, is converted to a RAS-ON active form through the interactive cooperation of GEFs and SOS1 interactive cooperation to trigger cancer cell growth and survival via RAF and PI3K activation. Conversely, the GTP-bound RAS-ON active form is converted to a GDP-bound inactive SWI/SWII RAS form by GAPs and NF1 acquisition of KRAS protein.

### 6.3 Combination of KRAS mutant inhibitor and SOS1 inhibitor for the KRAS mutant solid tumor treatment

Although KRAS(G12C) inhibitors have been spotlighted as an important targeted option for KRAS-mutant NSCLC, responses can be limited by adaptive pathway reactivation and acquired resistance. Accordingly, combination strategies that target multiple nodes of the RAS pathway are being explored to prolong and deepen responses in lung cancer. For example, co-targeting KRAS(G12C) together with upstream KRAS activators such as SOS1 has been proposed as a rational approach to suppress signaling rebound; some supporting preclinical evidence has been generated in non-lung models (72), and its relevance to NSCLC should be interpreted as a mechanistic rationale that motivates lung-focused validation and clinical translation.

### 6.4 Development of new type KRAS anti-cancer agents combined with DDR1

New anticancer treatment is needed for drug-resistant patients and KRAS-targeting mutants that are not treatable, such as KRAS G12V and G12D mutant patients. As an alternative to the existing KRAS inhibitor’s mechanism and resistance, new selective drugs to selectively target DDR1 that are known to applicable for diverse KRAS mutant cancer species have been considered. DDR1 belongs to a RTK of DDR family. DDR1 structure is constructed with intracellular domain (ID) containing kinase domain (KD), TM domain and extracellular domain containing two independent Discoidin and Discoidin-like domains (73).

DDR1 recognizes its ligand, a component of the extracellular matrix. After DDR1 binds to collagen, its KD located on the ID region is activated and autophosphorylated to transduce its signal. DDR1 activates Ras-Raf-ERK, PI3K-Akt and NF-κB signaling transduction to activate NF-κB/p62-NRF2 signaling, leading to cancer growth via cancer cell macropinocytosis and mitochondrial biosynthesis. DDR1 acts synergistically with the Notch receptor signaling pathway to upregulate cell proliferation, adhesion, migration and matrix remodeling, thereby enhancing cancer growth and metastatic potential. Currently, DDR1 inhibitors have 2 different acting mechanisms of (1) Type-I pharmacophore inhibitors that interfere with ATP binding to the ATP-binding site of DDR1 KD. (2) Type-II pharmacophore inhibitors recognize the DDR1 allosteric domain exposed when ATP binding site is in inactive conformation and thus maintain the inactive form state of DDR1 (74). DDR1 is known to modulate the immune cell infiltration, leading to anticancer modulation of breast cancer. In the DDR1-overexpressed tumor cells, infiltration of cytotoxic T cells is downregulated but reduction in cancer DDR1 expression shows anti-cancer efficacy by cytotoxic T cells. DDR1 has long been targeted to therapeutically compensate the diverse types of KRAS mutations. Therefore, the identification and development of first-in-class selective DDR1 inhibitors is the current stream of future new drugs (75). For example, combination therapy of inhibitors of PAN-KRAS mutants with DDR1-targeted inhibitors is also important. Recently, AstraZeneca UK, CEO Dr. Lisa Anson and Pfizer CSO Dr Richard Armer founded the Redx Co. to develop the DDR1 inhibitors. Selective DDR1 inhibitors can target the fibrosis regulation and provide synergistic effects to G12X (C, V, D) mutants in combination therapy with anti-fibrosis therapeutics.

## 7 Alternative therapeutic CSC targeting by TM protein SMO as the HHR

For alternative therapy, the TM protein SMO as a receptor of the HHR is a therapeutic target for the treatment of basal cell carcinoma patients, and thus, small molecular inhibitors of SMO have been developed for years. In addition, a gene for CSC’s self-renewal protein as a sonic hedgehog protein has been silenced by SMO siRNA transfection to treat BCC (76). CSC’s surface marker CD133 (promini-1) glycoprotein family known as a specific human hematopoietic progenitor cell marker is also targeted to kill brain tumors.

## 8 Miscellaneous tumor therapeutic targets

### 8.1 Serine hydroxymethyltransferase 1 silencing to block tumor DNA synthesis

Serine hydroxymethyltransferase 1 (SHMT1) enzyme can be targeted using siRNA-based gene delivery vector to inhibit DNA synthesis of tumor cells as an anti-tumor drug.

### 8.2 Carbonic anhydrase 9–targeting anticancer radioactive peptide

Carbonic anhydrase 9 (CAIX) is a TM metalloenzyme that regulates adhesion, proliferation, and intra/extracellular pH. CAIX-targeted blood peptide and Lutetium (Lu)-177 are anticancer drugs because CAIX can also be targeted for tumor diagnosis and tumor therapeutic radioactive peptide candidate (77). Lu-177 is a radionuclide used for targeted therapy and a theranostic isotope for neuroendocrine tumor therapy of metastatic castration-resistant prostate cancer (mCRPC) with its carrier/delivery molecules to selectively recognize tumor sites. Only Isotopia Ltd. company supplies LU-177 with the form in the presence of carrier-added molecule (CAM) and absence of CAM (78). CAIX is used as radiodiagnostic and therapeutic drug candidate for a kidney cancer subtype as a renal cell carcinoma known as clear cell renal cell carcinoma (ccRCC). ccRCC cancer cells show cells like clear soap bubbles and proliferate rapidly. A selective biomarker for hypoxic CSCs, the CAIX-targeting blood peptide and the radioisotope Lu-177 was used.

### 8.3 CLDN3-hyperexpressing solid tumor therapeutic antibody

Claudin-3 (CLDN3)–hyperexpressing solid tumor targeting drugs can also be developed using therapeutic antibody candidates for the treatment of claudin-3 overexpressing solid tumors. CLDNs are members of the family of tight junction proteins (79). CLDNs have two extracellular loops (ECL). Mammals have 27 CLDNs and humans have 26 CLDNs. Among the 27 known CLDNs, CLDN3 is a major and crucial component of the cell–cell junctions and aberrantly expressed in the invasive and metastatic tumor tissues. However, the cell–cell tight junctions must be disrupted during tumor cell metastasis. Therefore, CLDN3-targeting mAbs are considered as a first-in-class drug belonging to a tissue-agnostic type of agents. For example, ABN501 a fully human mAb has a high affinity at sub-nanomolar level to the native CLDN3 protein (80). ABN501 recognizes the ECL2 domain of claudin-3 and shows ADCC activity in CD16a (NK-92MI-CD16a)-expressing NK cells in cancer cells that expressing claudin-3 (80).

### 8.4 Importin-β–targeting and radiation therapy-enhancing biological agents of triple-negative breast cancer

Importin-β-targeting triple-negative breast cancer (TNBC) treatment drug and radiotherapy efficacy enhancing biological agents can be developed. For example, an importin-β-targeting inhibitor for TNBC therapy has been developed as a radio-enhancer for cancer therapy (81). Intractable tumors such as TNBC, SCLC, and NSCLC can be explored for novel type anti-tumor agents. Combination treatment with radioisotope therapy, targeted therapy, chemotherapy and immunotherapy against intractable tumors causes adverse side effects, hence radiotherapy efficacy-enhancing biological agents are emerging options. TNBC type are especially the cases. Combination treatment with radioisotope therapy increases mitochondrial ROS genesis and normal cell death. Then, biological agents that enhance the efficacy of radiotherapy have emerged therapeutic agents, emerged as therapeutic agents.

### 8.5 Anticancer antibiotics

Negatively charged phosphatidylserine present on the inner leaflet of the membrane can be exposed to the cell surface of the tumor outer membrane by the flip-flop process, and the flip-flopped negatively charged phosphatidylserine can interact with cationic anticancer peptides via electrostatic attraction. The development of anticancer peptides using tumor cell properties represents tumor cell membrane-targeting anticancer drugs. Anticancer peptides, as the first line defense molecules, consist of 10–50 amino acids with positively charged (+2-+11) amphipathic amino acid polymers that form various secondary structures with α-helix, β-sheet, or extended/random coils, but α-helix structures are predominant. To date, more than 3,000 anticancer peptides have been reported. Among them, 20 peptides exhibit anticancer activities by targeting the tumor cell membrane. However, they are cytotoxic to normal cells with low biological and chemical stabilities, which limits to clinical trials. Therefore, to overcome the limitations of anticancer peptides, several strategies, including chemical modification, amino acid substitution, and D-form amino acid incorporation, have emerged. Chemotherapy of cancer leads to a decrease in granulocyte count, and consequently, infectious diseases should be prevented using combination therapy with antibiotics. Thus, as a new cancer therapeutic strategy, the development of L-form amino acids’ anticancer peptides without artificial modification of sequences can simultaneously solve both issues of antibiotic resistance and anticancer resistance. For example, mitochondrial-specific sequences have amphipathic structures similar to α-helix–structured antibiotics. Because mitochondrial-specific sequences resemble antibiotics, which have an amphipathic α-helical structure, candidate peptides derived from mitochondrial-specific sequences may show anticancer activity. Mitochondrial-derived peptides that show simultaneously dual anticancer/antibiotic activities can be discovered using the https://www.proteinatlas.org/search/subcell_location:Mitochon, etc., and https://medals.jp/elist/detail/300.html. Eukaryotic mitochondria have been formed from the evolutionary event in which primitive bacteria interacted with ancestor eukaryotes, evolved into small organelles of host cells via endosymbiosis. Mitochondria have transferred genetic elements into the host cell nucleus by the endosymbiotic gene transfer, and mitochondria themselves have only minimal genes (82). For intrinsic biological functions, the nucleus, endoplasmic reticulum, and peroxisome proteins synthesized in the cytoplasmic region have distinct targeting sequences to their destination organelles. Similarly, mitochondrial proteins synthesized in the cytoplasmic region have a mitochondrial targeting sequence (MTS) to translocate to the mitochondrial organelle. The MTS has 10–50 amino acids with positive charge, but they are different from proteins translocated to other small organelles, such as the nucleus, ER, and peroxisome in their amphipathic and α-helix structures.

## 9 Recent ICI-based treatment for esophageal, gastric and colon cancers

ICI for unresectable advanced recurrent cancer has been widely proven in the treatment of esophageal cancer. The efficacy of Nivolumab (anti-PD-1 antibody) therapy compares with standard chemotherapy (paclitaxel or docetaxel) in patients with recurrent or advanced esophageal squamous cell carcinoma in a phase III randomized clinical trial via a multicenter, randomized and open-labeled phase 3 trial (ATTRACTION-3) study (ClinicalTrials.gov, NCT02569242) (83), demonstrating the priority of immunotherapy. Nivolumab significantly improved OS and safety compared with chemotherapy. Nivolumab therapy has been documented for second-line chemotherapy for esophageal cancer. An open-label and randomized phase III clinical study (KEYNOTE-181, ClinicalTrials.gov NCT02564263) compares Pembrolizumab (anti-PD-1 antibody) therapy with chemotherapy (paclitaxel, docetaxel, or Irinotecan) in patients with advanced/metastatic squamous cell carcinoma or adenocarcinoma of the esophagus (84). Pembrolizumab prolonged OS and was documented for second-line therapy for metastatic esophageal cancer patients. A randomized and placebo-controlled phase 3 clinical study (KEYNOTE-590, ClinicalTrials.gov, NCT03189719) examined the combined Pembrolizumab with chemotherapy [platinum-based cisplatin plus **F**luoropyrimidine (5-fluorouracil, 5-FU)] in patients with advanced esophageal and junction cancer. Efficacy in esophageal squamous cell carcinoma and adenocarcinoma suggests the combination therapy as a promising care (85), as recommended for the first-line chemotherapy. Neoadjuvant chemotherapy with Pembrolizumab plus chemotherapy has been suggested as a promising option for esophageal cancer. A phase III clinical trial (CheckMate 648, ClinicalTrials.gov, NCT03143153) examines a combination therapy of Nivolumab + chemotherapy and Nivolumab plus Ipilimumab (anti-CTLA-4 antibody) in advanced esophageal squamous cell carcinoma patients (86). Nivolumab and chemotherapy or Nivolumab plus Ipilimumab therapy is recommended as first-line chemotherapy for esophageal cancer (87). Unfortunately, PD-1 antibody is still uncertain in Nivolumab or Pembrolizumab combined therapy with chemotherapy (88).

Cancer immunotherapy in advanced and recurrent gastric cancers has been tried using ICI and ICI combination therapies. Therapeutic effect depends on the levels of PD-L1 and PD-L1 expression and is related to HER2-positive gastric cancer. Tyrosine kinase inhibitor Lenvatinib combined Pembrolizumab chemotherapy has been explored for HER2-negative gastric cancer. Anti-PD-L1 antibody Durvalumab has been used in advance gastric cancer and esophagogastric junction cancer (89). In a multicenter randomized and international phase III study (ATTRACTION-2) in advanced or metastatic gastric cancer patients, Nivolumab monotherapy was compared with placebo in chemotherapy resistant or progressed patients (90). Nivolumab therapy has been used as a back-line treatment for gastric cancer. In a phase III trial (ATTRACTION-4, ClinicalTrials.gov, NCT02746796), combined Nivolumab with chemotherapy is effective rather than conventional chemotherapy (91). In an international phase III multicenter randomized clinical trial (CheckMate649, ClinicalTrials.gov, NCT02872116), Nivolumab plus chemotherapy in advanced gastric or gastroesophageal junction cancer (92) has been suggested as a first-line chemotherapy for HER2-negative gastric cancer. In a randomized phase III trial (ATTRACTION-5, Clinical trial information: NCT03006705), Nivolumab has been used as adjuvant chemotherapy for gastric cancer. In a multicenter randomized phase III study ((KEYNOTE-859, ClinicalTrials.gov, NCT03675737), the combination of Pembrolizumab and chemotherapy was effective for HER2-negative advanced gastric or gastroesophageal junction cancer (93). In a multicentre, randomized and double-blind phase 3 trial (KEYNOTE-859, ClinicalTrials.gov, NCT03675737), the combination of Pembrolizumab and chemotherapy is recommended as first-line chemotherapy for HER2-negative gastric cancer. Furthermore, Zolbetuximab is also effective against HER2-negative, CLDN-positive gastric cancer (94). In multicentre, double-blind and randomisedrandomized phase 3 study (KEYNOTE-585, NCT04250948), neoadjuvant and adjuvant Pembrolizumab therapy improved advanced gastric cancer (95).

In colon cancer, the effectiveness of ICI depends on the status of microsatellite instability. In the MSI-h group, ICI-based treatment has been proven. Efficacy of colon cancer immunotherapy depends on the MSI (microsatellite instability). In the treatment of colorectal cancer, the effectiveness of ICI differs significantly depending on the status of microsatellite instability. In the MSI-h group, high efficacy of ICI has been proven, whereas efficacy of ICI is negative in the MSS group. As a new development, the efficacy of ICI as neoadjuvant chemotherapy in the MSI-h group is being explored. Among colon cancers, MSI-high or dMMR (DNA Mismatch Repair deficiency) colon cancers occupy 15%. Only 5% among colon cancers occupies metastatic colon cancers that are characterized by TILs (tumor-infiltrating lymphocytes) with activated PD-1/PD-L1 pathway, therefore, enhancing ICI effect (96). In a randomized, open-label, phase III study (KEYNOTE-177, ClinicalTrials.gov, NCT02563002), Pembrolizumab monotherapy compared with chemotherapy in patients with untreated advanced MSI-high/dMMR colon cancer (97). In a multicenter, non-randomized and single-armed phase II study (CheckMate142, ClinicalTrials.gov, NCT02060188), Nivolumab and Nivolumab plus Ipilimumab in patients with MSI-H/dMMR advanced colon cancer have a clinical benefit (98). ICI-based neoadjuvant chemotherapy may be highly effective for dMMR colon cancer. For example, Nivolumab plus Ipilimumab therapy has been recognized for dMMR colon cancer (99), and Nivolumab plus Relatlimab, an anti-LAG-3 antibody, has been recognized for dMMR colon cancer (100). In a phase II trial, anti-PD-1 Dostarlimab has been used as a neoadjuvant treatment for dMMR colon cancer (101). Biomarker-based ICI treatment is also another strategy because biomarkers accurately reflect the cancer immune status or the therapeutic effect of cancer immunotherapy. Prior to ICI-based treatment, molecular targeted therapy to HER2 using Trastzumab was introduced for HER2-positive gastric cancer. Zolbetuximab is effective for Claudin-positive gastric cancer in clinical practice during PD-L1 expression. ICI-based treatment can also be applied to HER2 positive cancer because multiple biomarkers are associated with successful chemotherapy in cancer.

## 10 Conclusion and future prospects

The present review comprehensively overviews ICI-based therapies in cancer, highlighting both combinatory and alternative strategies to enhance anti-tumor immune responses. The mechanisms of resistance, the role of the TME, and the integration of biomarkers and targeted therapies such as receptor tyrosine kinase inhibitors and approaches against cancer stem cells have been discussed with recent clinical advances, challenges in ICI efficacy, and future immunotherapies in various human cancers. For summary, recent conceptual approaches known as alternative and combination therapies have largely and compensatively been replaced by conventional therapy-resistant tumors. In the present perspectives and reviews, the current examples have been listed with their reasonable comparison. A recent immunotherapy termed anti-PD-l/PD-L1 therapy has long been performed using anti-cancer ICI or anti-cancer blocking drugs. To overcome the hurdles of ineffective ICI in NSCLC, a key oncogenic driver, EGFR, was analyzed for its gene mutant TKIs. Then, the successfully combined drugs of next-generation allosteric inhibitors have been designated against the EGRF inhibitor-resistant NSCLC. From the mutation information, their mutation-specific inhibitors have been developed to treat NSCLC and related cancer cells with the inhibitors. Expectedly, KRAS G12C-targeted inhibitors overcome the previous issues of the existing KRAS mutant inhibitors and their combination therapies. Similarly, ALK mutation in NSCLC has ALK mutation-specific inhibitors. ROS1 and RTK mutation, BRAF mutation, NTRK mutation, MET gene exon-14 skipping mutation, RET fusion mutation, EGFR-2 (HER2) mutation, and KRAS have also been discovered in NSCLC or other related cancers such as gastric, gastroesophageal adenocarcinoma, and breast cancer. From a prospective viewpoint, emerging therapeutic CSC can also be targeted by the HH pathway receptors. In addition, solid tumors can be easily targeted by a variety of emerging approaches, such as SHMT1 gene silencing, CAIX-targeting radioactive peptides, CLDN3-specific antibodies and importin-β targets, and radiation-enhancing biologics.

## Glossary

ABC: ATP-binding cassette
ADC: antibody–drug conjugate
ADCC: antibody-dependent cellular cytotoxicity
ALK: anaplastic lymphoma kinase
BCC: basal cell carcinoma
BCRP: breast cancer resistance protein
BMS: Bristol Myers Squibb
BRAF: B-Raf proto-oncogene, serine/threonine kinase
CAIX: carbonic anhydrase IX
CAM: carrier-added molecule
ccRCC: clear cell renal cell carcinoma
CLDN3: claudin-3
CNS: central nervous system
CSC: cancer stem cell(s)
CSCC: cutaneous squamous cell carcinoma
ctcDNA: circulating tumor cell DNA
ctDNA: circulating tumor DNA
CTLA-4: cytotoxic T-lymphocyte–associated protein 4
DDR: discoidin domain receptor
DOR: duration of response
ecDNA: extrachromosomal DNA
ECL: extracellular loop(s)
ECM: extracellular matrix
EGFR: epidermal growth factor receptor
EGFRmut: EGFR mutation (mutant EGFR)
EMA: European Medicines Agency
EML4: echinoderm microtubule-associated protein-like 4
ER: endoplasmic reticulum
ERBB2: Erb-B2 receptor tyrosine kinase 2 (HER2)
Exon20ins: EGFR exon 20 insertion(s)
FGFR: fibroblast growth factor receptor
HDAC: histone deacetylase
HHR: Hedgehog (HH) pathway receptor
HNC: head and neck cancer
IC: immune checkpoint
ICI: immune checkpoint inhibitor
ID: intracellular domain
ILD: interstitial lung disease
KD: kinase domain
Kd: dissociation constant
KRAS: Kirsten rat sarcoma viral oncogene homolog
MAA: marketing authorization application
mAb: monoclonal antibody
MCC: Merkel cell carcinoma
mCRPC: metastatic castration-resistant prostate cancer
MDR: multidrug resistance
MDSCs: myeloid-derived suppressor cells
MET: MET proto-oncogene, receptor tyrosine kinase
METex14: MET exon 14 skipping alteration
MHC-I: major histocompatibility complex class I
MTS: mitochondrial targeting sequence
NCI: National Cancer Institute
NDA: new drug application
NGS: next-generation sequencing
NSCLC: non-small cell lung cancer
NTRK: neurotrophic tyrosine receptor kinase
NTRK1: neurotrophic tyrosine receptor kinase 1
ORR: objective response rate
OS: overall survival
PD-1: programmed cell death protein 1
PD-L1: programmed death-ligand 1
PFS: progression-free survival
P-gp: P-glycoprotein
RET: rearranged during transfection
ROS1: ROS proto-oncogene 1, receptor tyrosine kinase
RR: response rate
RTK: receptor tyrosine kinase(s)
SCC: squamous cell carcinoma
SCLC: small cell lung cancer
Shh: Sonic hedgehog
SHMT1: serine hydroxymethyltransferase 1
SHP-2: Src homology region 2-containing protein tyrosine phosphatase-2 (PTPN11)
SLC: solute carrier
SMO: Smoothened
SOS1: SOS Ras/Rac guanine nucleotide exchange factor 1
TCI: T-cell infiltration
TKI: tyrosine kinase inhibitor
TM: transmembrane
TMB: tumor mutational burden
TME: tumor microenvironment
TNBC: triple-negative breast cancer

## References

[1] Sung H Ferlay J Siegel RL Laversanne M Soerjomataram I Jemal A . Global cancer statistics 2020: GLOBOCAN estimates of incidence and mortality worldwide for 36 cancers in 185 countries. CA: A Cancer J For Clin. (2021). doi: 10.3322/caac.21660 33538338

[2] Teng MW Galon J Fridman WH Smyth MJ . From mice to humans: Developments in cancer immunoediting. J Clin Invest. (2015) 125:3338–46. doi: 10.1172/jci80004 26241053 PMC4588291

[3] Zaretsky JM Garcia-Diaz A Shin DS Escuin-Ordinas H Hugo W Hu-Lieskovan S . Mutations associated with acquired resistance to PD-1 blockade in melanoma. N Engl J Med. (2016) 375:819–29. doi: 10.1056/NEJMoa1604958 27433843 PMC5007206

[4] Peng XX Yu R Wu X Wu SY Pi C Chen ZH . Correlation of plasma exosomal microRNAs with the efficacy of immunotherapy in EGFR/ALK wild-type advanced non-small cell lung cancer. J Immunother Cancer. (2020) 8:e000376. doi: 10.1136/jitc-2019-000376 31959728 PMC7057418

[5] Hofman P . Liquid biopsy for lung cancer screening: Usefulness of circulating tumor cells and other circulating blood biomarkers. Cancer Cytopathol. (2021) 129:341–6. doi: 10.1002/cncy.22367 33007153

[6] National Cancer Institute at the National Institutes of Health . Available online at: https://www.cancer.gov/news-events/cancer-currents-blog/2020/fda-foundation-one-cancer-liquid-biopsy-expanded-approval (Accessed October 27, 2021).

[7] Horn L Whisenant JG Wakelee H Reckamp KL Qiao H Leal TA . Monitoring therapeutic response and resistance: Analysis of circulating tumor DNA in patients with ALK+ lung cancer. J Thorac Oncol. (2019) 14:1901–11. doi: 10.1016/j.jtho.2019.08.003 31446141 PMC6823161

[8] Schwartzberg LS Horinouchi H Chan D Chernilo S Tsai ML Isla D . Liquid biopsy mutation panel for non-small cell lung cancer: Analytical validation and clinical concordance. NPJ Precis Oncol. (2020) 4:15. doi: 10.1038/s41698-020-0118-x 32596507 PMC7314769

[9] Li Z Liu F Wu S Ding S Chen Y Liu J . Research progress on the drug resistance of ALK kinase inhibitors. Curr Med Chem. (2022) 29:2456–75. doi: 10.2174/0929867328666210806120347 34365942

[10] Sun JY Zhang D Wu S Xu M Zhou X Lu XJ . Resistance to PD-1/PD-L1 blockade cancer immunotherapy: Mechanisms, predictive factors, and future perspectives. biomark Res. (2020) 8:1–0. doi: 10.1186/s40364-020-00212-5 32864132 PMC7450549

[11] Ishida Y Agata Y Shibahara K Honjo T . Induced expression of PD-1, a novel member of the immunoglobulin gene superfamily, upon programmed cell death. EMBO J. (1992) 11:3887–95. doi: 10.1002/j.1460-2075.1992.tb05481.x 1396582 PMC556898

[12] Le Mercier I Lines JL Noelle RJ . Beyond CTLA-4 and PD-1, the generation Z of negative checkpoint regulators. Front Immunol. (2015) 6:418. doi: 10.3389/fimmu.2015.00418 26347741 PMC4544156

[13] Postow MA Sidlow R Hellmann MD . Immune-related adverse events associated with immune checkpoint blockade. N Engl J Med. (2018) 378:158–68. doi: 10.1056/NEJMra1703481 29320654

[14] Lawrence MS Stojanov P Polak P Kryukov GV Cibulskis K Sivachenko A . Mutational heterogenicity in cancer and the search for new cancer-associated genes. Nature. (2013) 499:214–8. doi: 10.1038/nature12213 23770567 PMC3919509

[15] Robert C Ribas a Wolchok JD Hodi FS Hamid O Kefford R . Anti-programmed-deathreceptor-1 treatment with pembrolizumab in ipilimumab-refractory advanced melanoma: A randomised dose-comparison cohort of a phase 1 trial. Lancet Lond Engl. (2014) 384:1109–17. doi: 10.1016/s0140-6736(14)60958-2 25034862

[16] Oro-Ayude M Suh-Oh HJ Sacristán-Santos V Vázquez-Bartolomé P Flórez Á . Nivolumab for metastatic cutaneous squamous cell carcinoma. Case Rep Dermatol. (2020) 12:37–41. doi: 10.1159/000505478 32595466 PMC7315375

[17] Liu K Sun Q Liu Q Li H Zhang W Sun C . Focus on immune checkpoint PD-1/PD-L1 pathway: New advances of polyphenol phytochemicals in tumor immunotherapy. BioMed Pharmacother. (2022) 154:113618. doi: 10.1016/j.biopha.2022.113618 36055113

[18] Zhang Q Yang C Gao X Dong J Zhong C . Phytochemicals in regulating PD-1/PD-L1 and immune checkpoint blockade therapy. Phytother Res. (2024) 38:776–96. doi: 10.1002/ptr.8082 38050789

[19] Zhu W Wang JP Meng QZ Zhu F Hao XF . MiR-142-5p reverses the resistance to gefitinib through targeting HOXD8 in lung cancer cells. Eur Rev Med Pharmacol Sci. (2020) 24:4306–13. doi: 10.26355/eurrev_202004_21011 32373967

[20] Tan CS Kumarakulasinghe NB Huang YQ Ang YLE Choo JR Goh BC . Third generation EGFR TKIs: Current data and future directions. Mol Cancer. (2018) 17:29. doi: 10.1186/s12943-018-0778-0 29455654 PMC5817792

[21] Camidge DR Pao W Sequist LV . Acquired resistance to TKIs in solid tumours: Learning from lung cancer. Nat Rev Clin Oncol. (2014) 11:473–81. doi: 10.1038/nrclinonc.2014.104 24981256

[22] Rosell R Moran T Queralt C Porta R Cardenal F Camps C . Screening for epidermal growth factor receptor mutations in lung cancer. N Engl J Med. (2009) 361:958–67. doi: 10.1056/nejmoa0904554 19692684

[23] Lazzari C Gregorc V Karachaliou N Rosell R Santarpia M . Mechanisms of resistance to osimertinib. J Thorac Dis. (2020) 12:2851–8. doi: 10.21037/jtd.2019.08.30 32642198 PMC7330330

[24] Gray JE Ahn MJ Oxnard GR Shepherd FA Imamura F Cheng Y . Early clearance of plasma epidermal growth factor receptor mutations as a predictor of outcome on osimertinib in advanced non-small cell lung cancer; Exploratory analysis from AURA3 and FLAURA. Clin Cancer Res. (2023) 29:3340–51. doi: 10.1158/1078-0432.CCR-22-3146 37379430

[25] Mack PC Redman MW Moon J Goldberg SB Herbst RS Melnick MAC . Residual circulating tumor DNA (CtDNA) after two months of therapy to predict progression-free and overall survival in patients treated on S1403 with afatinib +/- cetuximab. J Clin Oncol. (2020) 38:9532. doi: 10.1200/jco.2020.38.15_suppl.9532 42148471

[26] Socinski MA Jotte RM Cappuzzo F Orlandi F Stroyakovskiy D Nogami N . Atezolizumab for first-line treatment of metastatic nonsquamous NSCLC. N Engl J Med. (2018) 378:2288–301. doi: 10.1056/nejmoa1716948 29863955

[27] Reck M Mok TSK Nishio M Jotte RM Cappuzzo F Orlandi F . Atezolizumab plus bevacizumab and chemotherapy in non-small-cell lung cancer (IMpower150): Key subgroup analyses of patients with EGFR mutations or baseline liver metastases in a randomised, open-label phase 3 trial. Lancet Respir Med. (2019) 7:387–401. doi: 10.1016/s2213-2600(19)30084-0 30922878

[28] Du X Yang B An Q Assaraf YG Cao X Xia J . Acquired resistance to third-generation EGFR-TKIs and emerging next-generation EGFR inhibitors. Cell. (2021) 2:1–17. doi: 10.1016/j.xinn.2021.100103 34557754 PMC8454558

[29] Food and Drugs Administration . FDA approves osimertinib as adjuvant therapy for non-small cell lung cancer with EGFR mutations. Available online at: https://www.fda.gov/drugs/resources-information-approved-drugs/fda-approves-osimertinib-adjuvant-therapy-non-small-cell-lung-cancer-egfr-mutations (Accessed February 16, 2024).

[30] Shapira A Livney YD Broxterman HJ Assaraf YG . Nanomedicine for targeted cancer therapy: Towards the overcoming of drug resistance. Drug Resist Update. (2011) 14:150–63. doi: 10.1016/j.drup.2011.01.003 21330184

[31] Chen KG Sikic BI . Molecular pathways: Regulation and therapeutic implications of multidrug resistance. Clin Cancer Res. (2012) 18:1863–9. doi: 10.1158/1078-0432.ccr-11-1590 22344233 PMC3359695

[32] To C Beyett TS Jang J Feng WW Bahcall M Haikala HM . An allosteric inhibitor against the therapy-resistant mutant forms of EGFR in non-small cell lung cancer. Nat Cancer. (2022) 3:402–17. doi: 10.1038/s43018-022-00351-8 35422503 PMC9248923

[33] Song Z Ren G Hu L Wang X Song J Jia Y . Two case reports of non-small cell lung cancer patients harboring acquired EGFR T790M-cis-C797S benefit from immune checkpoint inhibitor combined with platinum-based doublet chemotherapy. Ann Transl Med. (2022) 10:719. doi: 10.21037/atm-22-2436 35845537 PMC9279785

[34] Corvaja C Passaro A Attili I Aliaga PT Spitaleri G Signore D . Advancements in fourth-generation EGFR TKIs in EGFR-mutant NSCLC: Bridging biological insights and therapeutic development. Cancer Treat Rev. (2024) 130:102824. doi: 10.1016/j.ctrv.2024.102824 39366135

[35] Zhao HY Xi XX Xin M Zhang SQ . Overcoming C797S mutation: The challenges and prospects of the fourth-generation EGFR-TKIs. Bioorg Chem. (2022) 128:106057. doi: 10.1016/j.bioorg.2022.106057 35964503

[36] Brazel D Nagasaka M . MARIPOSA: Can amivantamab and lazertinib replace osimertinib in the front-line setting? Lung Cancer (Auckl). (2024) 15:41–7. doi: 10.2147/LCTT.S453974 38633373 PMC11021860

[37] Meyer ML Fitzgerald BG Paz-Ares L Cappuzzo F Jänne PA Peters S . New promises and challenges in the treatment of advanced non-small-cell lung cancer. Lancet. (2024) 404:803–22. doi: 10.1016/S0140-6736(24)01029-8 39121882

[38] Moes-Sosnowska J Szpechcinski A Chorostowska-Wynimko J . Clinical significance of TP53 alterations in advanced NSCLC patients treated with EGFR, ALK and ROS1 tyrosine kinase inhibitors: An update. Tumour Biol. (2024) 46:S309–25. doi: 10.3233/TUB-230034 37840519

[39] Shaw AT Ou SH Bang YJ Camidge DR Solomon BJ Salgia R . Crizotinib in ROS1-rearranged non-small-cell lung cancer. N Engl J Med. (2014) 371:1963–71. doi: 10.1056/NEJMoa1406766 25264305 PMC4264527

[40] Dong S Yousefi H Savage IV Okpechi SC Wright MK Matossian MD . Ceritinib is a novel triple negative breast cancer therapeutic agent. Mol Cancer. (2022) 21:138. doi: 10.1186/s12943-022-01601-0 35768871 PMC9241294

[41] Desai AV Robinson GW Gauvain K Basu EM Macy ME Maese L . Entrectinib in children and young adults with solid or primary CNS tumors harboring NTRK, ROS1, or ALK aberrations (STARTRK-NG). Neuro Oncol. (2022) 24:1776–89. doi: 10.1093/neuonc/noac087 35395680 PMC9527518

[42] Weng CD Liu KJ Jin S Su JW Yao YH Zhou CZ . Triple-targeted therapy of dabrafenib, trametinib, and osimertinib for the treatment of the acquired BRAF V600E mutation after progression on EGFR-tyrosine kinase inhibitors in advanced EGFR-mutated non-small cell lung cancer patients. Transl Lung Cancer Res. (2024) 13:2538–48. doi: 10.21037/tlcr-24-358 39507030 PMC11535828

[43] Bouffet E Hansford JR Garrè ML Hara J Plant-Fox A Aerts I . Dabrafenib plus trametinib in pediatric glioma with BRAF V600 mutations. N Engl J Med. (2023) 389:1108–20. doi: 10.1056/NEJMoa2303815 37733309

[44] Smith MR Dixon CB Wang Y Liu Y D'Agostino RJ Ruiz J . Targeting NTRK1 enhances immune checkpoint inhibitor efficacy in NTRK1 wild-type non-small cell lung cancer. Cancer Res. (2024) 84:4002–16. doi: 10.1158/0008-5472.CAN-24-0658 39250241 PMC11611666

[45] Liu F Wei Y Zhang H Jiang J Zhang P Chu Q . NTRK fusion in non-small cell lung cancer: Diagnosis, therapy, and TRK inhibitor resistance. Front Oncol. (2022) 12:864666. doi: 10.3389/fonc.2022.864666 35372074 PMC8968138

[46] Cancer Genome Atlas Research Network . Comprehensive molecular profiling of lung adenocarcinoma. Nature. (2014) 511:543–50. doi: 10.1038/nature13385 25079552 PMC4231481

[47] European Medicine Agency . Tepmetko, EMA/778167/2021. Available online at: https://www.ema.europa.eu/en/medicines/human/EPAR/tepmetko.

[48] U.S. Food and Drug Administration . Tepmekto. (2021).

[49] Reck M Remon J Hellmann MD . First-line immunotherapy for non-small-cell lung cancer. J Clin Oncol. (2022) 40:586–97. doi: 10.1200/jco.21.01497 34985920

[50] Liu T Wu S Fang W Li H Su L Qi G . Identifying optimal first-line immune checkpoint inhibitors-based regiments for advanced non-small cell lung cancer without oncogenic driver mutations: A systematic review and network meta-analysis. PloS One. (2023) 18:e0283719. doi: 10.1371/journal.pone.0283719 37071610 PMC10112813

[51] Mazieres J Drilon A Lusque A Mhanna L Cortot AB Mezquita L . Immune checkpoint inhibitors for patients with advanced lung cancer and oncogenic driver alterations: results from the IMMUNOTARGET registry. Ann Oncol. (2019) 30:1321–8. doi: 10.1093/annonc/mdz167 31125062 PMC7389252

[52] Sabari JK Leonardi GC Shu CA Umeton R Montecalvo J Ni A . PD-L1 expression, tumor mutational burden, and response to immunotherapy in patients with MET exon 14 altered lung cancers. Ann Oncol. (2018) 29:2085–91. doi: 10.1093/annonc/mdy334 30165371 PMC6225900

[53] Schrock AB Frampton GM Suh J Chalmers ZR Rosenzweig M Erlich RL . Characterization of 298 patients with lung cancer harboring MET exon 14 skipping alterations. J Thorac Oncol. (2016) 11:1493–502. doi: 10.1016/j.jtho.2016.06.004 27343443

[54] Febres-Aldana CA Vojnic M Odintsov I Zhang T Cheng R Beach CZ . Pan-cancer analysis of oncogenic MET fusions reveals distinct pathogenomic subsets with differential sensitivity to MET-targeted therapy. Cancer Discov. (2025) 15:1141–58. doi: 10.1158/2159-8290.CD-24-0417 39965191 PMC12133426

[55] Paik PK Iams WT Husain H O'Hara RM Jr Adewusi E Le X . Tepotinib in patients with MET exon 14 skipping non-small cell lung cancer. Cancer Treat Rev. (2025) 139:102990. doi: 10.1016/j.ctrv.2025.102990 40738075

[56] Subbiah V Velcheti V Tuch BB Ebata K Busaidy NL Cabanillas ME . Selective RET kinase inhibition for patients with RET-altered cancers. Ann Oncol. (2018) 29:1869–76. doi: 10.1093/annonc/mdy137 29912274 PMC6096733

[57] Gálffy G Morócz É Korompay R Hécz R Bujdosó R Puskás R . Targeted therapeutic options in early and metastatic NSCLC-overview. Pathol Oncol Res. (2024) 30:1611715. doi: 10.3389/pore.2024.1611715 38605928 PMC11006988

[58] Park S Park S Kim TM Kim S Koh J Lim J . Resistance mechanisms of EGFR tyrosine kinase inhibitors, in EGFR exon 20 insertion-mutant lung cancer. Eur J Cancer. (2024) 208:114206. doi: 10.1016/j.ejca.2024.114206 38981315

[59] Jani V Sonavane U Joshi R . Insight into structural dynamics involved in activation mechanism of full length KRAS wild type and P-loop mutants. Heliyon. (2024) 10:e36161. doi: 10.1016/j.heliyon.2024.e36161 39247361 PMC11379609

[60] Cox AD Der CJ . Undruggable KRAS": druggable after all. Genes Dev. (2025) 39:132–62. doi: 10.1101/gad.352081.124 39638567 PMC11789494

[61] Vasta JD Peacock DM Zheng Q Walker JA Zhang Z Zimprich CA . KRAS is vulnerable to reversible switch-II pocket engagement in cells. Nat Chem Biol. (2022) 18:596–604. doi: 10.1038/s41589-022-00985-w 35314814 PMC9135634

[62] Nokin MJ Mira A Patrucco E Ricciuti B Cousin S Soubeyran I . RAS-ON inhibition overcomes clinical resistance to KRAS G12C-OFF covalent blockade. Nat Commun. (2024) 15:7554. doi: 10.1038/s41467-024-51828-2 39215000 PMC11364849

[63] Higgins JP Carlisle JW Moniri NH Gupta S Oduah EI Leal T . Sotorasib for the treatment of locally advanced/metastatic non-small cell lung cancer. Future Oncol. (2025) 21:63–71. doi: 10.1080/14796694.2024.2430172 39601038 PMC11789721

[64] Negrao MV Spira AI Heist RS Jänne PA Pacheco JM Weiss J . Intracranial efficacy of adagrasib in patients from the KRYSTAL-1 trial with KRAS(G12C)-mutated non-small-cell lung cancer who have untreated CNS metastases. J Clin Oncol. (2023) 41:4472–7. doi: 10.1200/JCO.23.00046 37327468 PMC10553074

[65] Parums DV . Editorial: Recent approval of sotorasib as the first targeted therapy for KRAS G12C-mutated advanced non-small cell lung cancer (NSCLC). Med Sci Monit. (2022) 28:e938746. doi: 10.12659/MSM.938746 36317327 PMC9636839

[66] Ou SI Jänne PA Leal TA Rybkin II Sabari JK Barve MA . First-in-human phase I/IB dose-finding study of adagrasib (MRTX849) in patients with advanced KRAS(G12C) solid tumors (KRYSTAL-1). J Clin Oncol. (2022) 40:2530–8. doi: 10.1200/JCO.21.02752 35167329 PMC9362872

[67] Ostrem JM Shokat KM . Targeting KRAS G12C with covalent inhibitors. Annu Rev Cancer Biol. (2022) 6:49–64. doi: 10.1146/annurev-cancerbio-041621-012549 41139587

[68] Haddad SF Bouferraa Y Nair KG . Adagrasib in the treatment of colorectal cancer. Future Oncol. (2025) 21:2275–85. doi: 10.1080/14796694.2025.2524311 40619745 PMC12323406

[69] Blair HA . Sotorasib: first approval. Drugs. (2021) 81:1573–9. doi: 10.1007/s40265-021-01574-2 34357500 PMC8531079

[70] Awad MM Liu S Rybkin II Arbour KC Dilly J Zhu VW . Acquired resistance to KRASG12C inhibition in cancer. N Engl J Med. (2021) 384:2382–93. doi: 10.1056/NEJMoa2105281 34161704 PMC8864540

[71] Feng J Xiao X Xia X Min J Tang W Shi X . A pan-KRAS inhibitor and its derived degrader elicit multifaceted anti-tumor efficacy in KRAS-driven cancers. Cancer Cell. (2025) 3:209–49. doi: 10.1016/j.ccell.2025.07.006 40780213

[72] Plangger A Rath B Stickler S Hochmair M Lang C Weigl L . Cytotoxicity of combinations of the pan-KRAS SOS1 inhibitor BAY-293 against pancreatic cancer cell lines. Discov Oncol. (2022) 13:84. doi: 10.1007/s12672-022-00550-w 36048281 PMC9437170

[73] Kothiwale S Borza CM Lowe EW Jr Pozzi A Meiler J . Discoidin domain receptor 1 (DDR1) kinase as target for structure-based drug discovery. Drug Discov Today. (2015) 20:255–61. doi: 10.1016/j.drudis.2014.09.025 25284748 PMC4336622

[74] Borza CM Su Y Tran TL Yu L Steyns N Temple KJ . Discoidin domain receptor 1 kinase activity is required for regulating collagen IV synthesis. Matrix Biol. (2017) 57-58:258–71. doi: 10.1016/j.matbio.2016.11.009 27915093 PMC5329129

[75] Wu D Ding Z Lu T Chen Y Zhang F Lu S . DDR1-targeted therapies: current limitations and future potential. Drug Discov Today. (2024) 29:103975. doi: 10.1016/j.drudis.2024.103975 38580164

[76] Pandey S Lee M Lim J Park S Choung YH Kim JE . SMO-CRISPR-mediated apoptosis in CD133-targeted cancer stem cells and tumor growth inhibition. J Control Release. (2023) 357:94–108. doi: 10.1016/j.jconrel.2023.03.023 36931470

[77] Li C Hu Q Liang SH . CAIX-targeted PET imaging agents based on acetazolamide small molecule for clear cell renal cell carcinoma. Am J Nucl Med Mol Imaging. (2025) 15:37–43. doi: 10.62347/VHYY2134 40124762 PMC11929011

[78] Harris PE Zhernosekov K . The evolution of PRRT for the treatment of neuroendocrine tumors; what comes next? Front Endocrinol (Lausanne). (2022) 13:941832. doi: 10.3389/fendo.2022.941832 36387893 PMC9659917

[79] Beier LS Piontek J Piontek A Protze J Kobelt D Walther W . Claudin-targeted suicide gene therapy for claudin-overexpressing tumor cells by using modified Clostridium perfringens enterotoxin (CPE). Methods Mol Biol. (2022) 2521:173–88. doi: 10.1007/978-1-0716-2441-8_9 35732998

[80] Lee SH Choi J Hong SH Kim JS Lee SI Hong SG . Abstract 2653: ABN501, a novel anti-claudin-3 antibody, shows potent anti-cancer activity *in vitro* and *in vivo*. POSTER PRESENTATIONS - PROFFERED ABSTRACTS| APRIL 04 2023. Cancer Res. (2023) 83:2653. doi: 10.1158/1538-7445.AM2023-2653 36230740

[81] Wang T Ba X Zhang X Zhang N Wang G Bai B . Nuclear import of PTPN18 inhibits breast cancer metastasis mediated by MVP and importin beta2. Cell Death Dis. (2022) 13:720. doi: 10.1038/s41419-022-05167-z 35982039 PMC9388692

[82] Wang Z Zhang RX Zhang T He C He R Ju X . In situ proapoptotic peptide-generating rapeseed protein-based nanocomplexes synergize chemotherapy for cathepsin-B overexpressing breast cancer. ACS Appl Mater Interfaces. (2018) 10:41056–69. doi: 10.1021/acsami.8b14001 30387987

[83] Kato K Cho BC Takahashi M Okada M Lin CY Chin K . Nivolumab versus chemotherapy in patients with advanced esophageal squamous cell carcinoma refractory to previous chemotherapy (ATTRACTION-3): a multicentre, randomised, open-label, phase 3 trial. Lancet Oncol. (2019) 20:1506–17. doi: 10.1016/S1470-2045(19)30626-6 31582355

[84] Kojima T Shah MA Muro K Eric F Antoine A Hsu CH . Randomized phase III KEYNOTE-181 study of pembrolizumab versus chemotherapy in advanced esophageal cancer. J Clin Oncol. (2020) 38:4138–48. doi: 10.1200/JCO.20.01888 33026938

[85] Sun JM Shen L Shah MA Peter E Antoine A Doi T . Pembrolizumab plus chemotherapy versus chemotherapy alone for first-line treatment of advanced oesophageal cancer (KEYNOTE-590): a randomised, placebo-controlled, phase 3 study. Lancet. (2021) 398:759–71. doi: 10.1016/S0140-6736(21)01234-4 34454674

[86] Doki Y Ajani JA Kato K Jianming X Lucjan W Motoyama S . Nivolumab combination therapy in advanced esophageal squamous-cell carcinoma. N Engl J Med. (2022) 386:449–62. doi: 10.1056/NEJMoa2111380 35108470

[87] Kitagawa Y Ishihara R Ishikawa H Ito Y Oyama T Oyama T . Esophageal cancer practice guidelines 2022 edited by the Japan esophageal society: part 1. Esophagus. (2023) 20:343–72. doi: 10.1007/s10388-023-00993-2 36933136 PMC10024303

[88] Kelly RJ Ajani JA Kuzdzal J Zander T Van Cutsem E Piessen G . Adjuvant nivolumab in resected esophageal or gastroesophageal junction cancer. N Engl J Med. (2021) 384:1191–203. doi: 10.1056/NEJMoa2032125 33789008

[89] Janjigian YY Van Cutsem E Muro K Wainberg Z Al-Batran SE Hyung WJ . MATTERHORN: phase III study of durvalumab plus FLOT chemotherapy in resectable gastric/gastroesophageal junction cancer. Future Oncol. (2022) 18:2465–73. doi: 10.2217/fon-2022-0093 35535555

[90] Kang YK Boku N Satoh T Ryu MH Chao Y Kato K . Nivolumab in patients with advanced gastric or gastro-oesophageal junction cancer refractory to, or intolerant of, at least two previous chemotherapy regimens (ONO-4538-12, ATTRACTION-2): a randomised, double-blind, placebo-controlled, phase 3 trial. Lancet. (2017) 390:2461–71. doi: 10.1016/S0140-6736(17)31827-5 28993052

[91] Kang YK Chen LT Ryu MH Oh DY Oh SC Chung HC . Nivolumab plus chemotherapy versus placebo plus chemotherapy in patients with HER2-negative, untreated, unresectable advanced or recurrent gastric or gastro-oesophageal junction cancer (ATTRACTION-4): a randomised, multicentre, double-blind, placebo-controlled, phase 3 trial. Lancet Oncol. (2022) 23:234–47. doi: 10.1016/S1470-2045(21)00692-6 35030335

[92] Janjigian YY Shitara K Moehler M Garrido M Salman P Shen L . First-line nivolumab plus chemotherapy versus chemotherapy alone for advanced gastric, gastro-oesophageal junction, and oesophageal adenocarcinoma (CheckMate 649): a randomised, open-label, phase 3 trial. Lancet. (2021) 398:27–40. doi: 10.1016/S0140-6736(21)00797-2 34102137 PMC8436782

[93] Rha SY Oh DY Yañez P Bai Y Ryu MH Lee J . Pembrolizumab plus chemotherapy versus placebo plus chemotherapy for HER2-negative advanced gastric cancer (KEYNOTE-859): a multicentre, randomised, double-blind, phase 3 trial. Lancet Oncol. (2023) 24:1181–95. doi: 10.1016/S1470-2045(23)00515-6 37875143

[94] Shah MA Shitara K Ajani JA Bang YJ Enzinger P Ilson D . Zolbetuximab plus CAPOX in CLDN18.2-positive gastric or gastroesophageal junction adenocarcinoma: the randomized, phase 3 GLOW trial. Nat Med. (2023) 29:2133–41. doi: 10.1038/s41591-023-02465-7 37524953 PMC10427418

[95] Shitara K Rha SY Wyrwicz LS Oshima T Karaseva N Osipov M . Neoadjuvant and adjuvant pembrolizumab plus chemotherapy in locally advanced gastric or gastro-oesophageal cancer (KEYNOTE-585): an interim analysis of the multicentre, double-blind, randomised phase 3 study. Lancet Oncol. (2024) 25:212–24. doi: 10.1016/S1470-2045(23)00541-7 38134948

[96] Le DT Durham JN Smith KN Wang H Bartlett BR Aulakh LK . Mismatch repair deficiency predicts response of solid tumors to PD-1 blockade. Science. (2017) 357:409–13. doi: 10.1126/science.aan6733 28596308 PMC5576142

[97] Diaz LA Jr Shiu KK Kim TW Jensen BV Jensen LH Punt C . Pembrolizumab versus chemotherapy for microsatellite instability-high or mismatch repair-deficient metastatic colorectal cancer (KEYNOTE-177): final analysis of a randomised, open-label, phase 3 study. Lancet Oncol. (2022) 23:659–70. doi: 10.1016/S1470-2045(22)00197-8 35427471 PMC9533375

[98] André T Lonardi S Wong KYM Lenz HJ Gelsomino F Aglietta M . Nivolumab plus low-dose ipilimumab in previously treated patients with microsatellite instability-high/mismatch repair-deficient metastatic colorectal cancer: 4-year follow-up from CheckMate 142. Ann Oncol. (2022) 33:1052–60. doi: 10.1016/j.annonc.2022.06.008 35764271

[99] Chalabi M Verschoor YL Tan PB Balduzzi S Van Lent AU Grootscholten C . Neoadjuvant immunotherapy in locally advanced mismatch repair-deficient colon cancer. N Engl J Med. (2024) 390:1949–58. doi: 10.1056/NEJMoa2400634 38838311

[100] de Gooyer PGM Verschoor YL van den Dungen LDW Balduzzi S Marsman HA Geukes Foppen MH . Neoadjuvant nivolumab and relatlimab in locally advanced MMR-deficient colon cancer: a phase 2 trial. Nat Med. (2024) 30:3284–90. doi: 10.1038/s41591-024-03250-w 39278994 PMC11564102

[101] Cercek A Lumish M Sinopoli J Weiss J Shia J Lamendola-Essel M . PD-1 blockade in mismatch repair-deficient, locally advanced rectal cancer. N Engl J Med. (2022) 386:2363–76. doi: 10.1056/NEJMoa2201445 35660797 PMC9492301

